# Pentacoordinated Organotin(IV) Complexes as an Alternative in the Design of Highly Efficient Optoelectronic and Photovoltaic Devices: Synthesis and Photophysical Characterization

**DOI:** 10.3390/ijms24065255

**Published:** 2023-03-09

**Authors:** María Elena Sánchez Vergara, Elizabeth Gómez, Emiliano Toledo Dircio, José Ramón Álvarez Bada, Samuel Cuenca Pérez, José Miguel Galván Hidalgo, Arturo González Hernández, Simón Hernández Ortega

**Affiliations:** 1 Facultad de Ingeniería, Universidad Anáhuac México, Avenida Universidad Anáhuac 46, Col. Lomas Anáhuac, Huixquilucan 52786, Mexico; 2 Instituto de Química, Universidad Nacional Autónoma de México, Circuito Exterior s/n. C.U., Alcaldía Coyoacán, Ciudad de México 04510, Mexico

**Keywords:** organotin–Schiff bases, organotin(IV) complexes, pentacoordinated organotin, hybrid film, PEDOT:PSS, graphene, optical properties, electrical properties

## Abstract

The synthesis of four pentacoordinated organotin(IV) complexes prepared in a one-pot reaction from 2-hydroxy-1-naphthaldehyde, 2-amino-3-hydroxypyridine and organotin oxides is reported. The complexes were characterized by UV-Vis, IR, MS, ^1^H, ^13^C and ^119^Sn NMR techniques. The compound based on 2,2-diphenyl-6-aza-1,3-dioxa-2-stannanaphtho[1,2-h]pyrido[3,2-d]cyclononene revealed the formation of a monomeric complex with a distorted five-coordinated molecular geometry intermediate between the trigonal bipyramidal and square pyramidal. In order to find possible applications in photovoltaic devices, hybrid films of organotin(IV) complexes embedded in poly(3,4-ethylenedioxythiophene):poly(styrenesulfonate) (PEDOT:PSS) with graphene were deposited. The topographic and mechanical properties were examined. The film with the complex integrated into the cyclohexyl substituent has high plastic deformation, with a maximum stress of 1.69 × 10^7^ Pa and a Knoop hardness of 0.061. The lowest values of 1.85 eV for the onset gap and 3.53 eV for the energy gap were obtained for the heterostructure having the complex with the phenyl substituent. Bulk heterojunction devices were fabricated; these devices showed ohmic behavior at low voltages and a space-charge-limited current (SCLC) conduction mechanism at higher voltages. A value of 0.02 A was found for the maximum carried current. The SCLC mechanism suggests hole mobility values of between 2.62 × 10^−2^ and 3.63 cm^2^/V^.^s and concentrations of thermally excited holes between 2.96 × 10^18^ and 4.38 × 10^18^ m^−3^.

## 1. Introduction

Thin-film organic photovoltaic technology has been the subject of considerable attention because of the advantages it provides, such as light devices and low preparation cost [1,2,3,4]. Within this field, small-molecule heterojunction solar cells receive more attention because of their clear molecular structure, molecular weight and controllable material purity [5,6,7]. In small-molecule solar cells, the use of fullerenes and phthalocyanines has been widely reported [8,9]; nevertheless, there are a large number of small molecules that are also worthy of study. In this regard, organotin(IV) complexes may be a novel option. Organotin(IV) derivatives have been given special consideration because of the stable bonds formed by tin with carbon atoms, as well as with heteroatoms [10,11,12]. Particular consideration has been given to organotin complexes derived from Schiff bases, since these bases provide a wide variety of molecular structural conformations as ligands [13,14,15,16,17]. Moreover, complexes with Schiff bases show thermo- and photostability, are easy to synthesize and have the versatility to tune optical and optoelectronic properties through the chemical modulation of the ligands [18]. The optoelectronic activity of organotin(IV) complexes depends upon the number and nature of the radical groups linked to the metal ion, as well as on the anionic ligand [19]. Their azomethine C=N group presents an electron-withdrawing character that, combined with tin, results in “push-pull” molecules with non-linear optical and electrical properties [17,20,21,22,23], which may represent potential applications in optoelectronics. According to the push–pull model, photoinduced charge transfer is possible and usable in photovoltaic devices [21,24]. Organotin(IV) complexes have also been reported as sensitizers [25], PVC stabilizers [26], fungicides, catalysts, and active electroluminescent layers in organic light-emitting diodes (OLEDs) [27]. In addition, organotin(IV) complexes can be used in the manufacture of semiconductor films, in which their electrical conductivity can be increased by adding substituents to their molecular structure [28,29,30]. Charge transport is present in organotin(IV) films due to their π-conjugated structures and the presence of electronegative atoms and substituents coordinated to the tin atom [31].

Keeping the above in mind, and in order to broaden the study of different tin coordination structures, in this work, we report the synthesis of four pentacoordinated organotin(IV) complexes prepared in a one-pot reaction from 2-hydroxy-1-naphthaldehyde, 2-amino-3-hydroxypyridine and organotin(IV) oxides. This work is divided into three parts: the first one corresponds to the synthesis and characterization of the pentacoordinated organotin(IV) complexes; the second one refers to the fabrication and characterization of poly(3,4-ethylenedioxythiophene):poly(styrenesulfonate) (PEDOT:PSS)-graphene-organotin(IV) complex hybrid films, and the third one refers to the manufacture and electrical characterization of devices made from the hybrid films. It is important to mention that PEDOT:PSS is one of the best-known conjugate polymers that have proved their value in the development of electronic devices. PEDOT:PSS provides excellent optoelectronic functionalities, as highly transparent and electrochemically stable conducting films can be obtained from its doped state. The combination of PEDOT:PSS with chemically different structures, such as graphene, permits the improvement of their electrical properties and their use in various kinds of photovoltaic applications [32,33,34,35,36]. Because of their π-π stacking interactions, the conjugated aromatic chains of PEDOT:PSS can be stably fixed on graphene sheets without destroying the electronic structure of graphene [37]. The π-π stacking interactions between graphene and PEDOT:PSS results in enhanced electrical conductivity, chemical stability and thermoelectric performance [38]. In this work, PEDOT:PSS and graphene function as a hole-transport layer (HTL), while the organotin complexes operate as components of the active layer of the device. 

The behavior of organotin(IV) complexes is related to their π-conjugated structure, including the presence of electron-donating or electron-accepting substituents. This turns organotin(IV) complexes into anisotropic semiconductors with preferential conduction channels for charge transport. The novelty of this work is related to the synthesis of new pentacoordinated tin complexes and their use in hybrid films with optical and electrical properties.

## 2. Results and Discussion

### 2.1. Synthesis and Characterization of Pentacoordinated Organotin(IV) Complexes

**Scheme 1:**
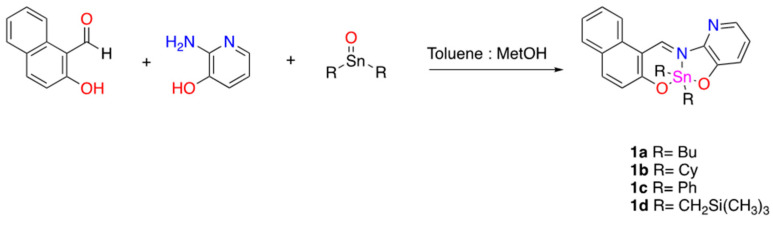
Synthetic strategy for the production of organotin(IV) complexes.

Complexes **1a**–**1d** were synthesized in a one-pot reaction. 2-Hydroxy-1-naphthaldehyde, 2-amino-3-hydroxypyridine and the corresponding organotin oxide in a stoichiometric ratio 1:1:1 were refluxed using a solvent mixture of toluene–methanol (80:20 *v/v*), as shown in Scheme 1. The reaction proceeded with yields of 57 to 97%. All complexes were isolated as red solids and were soluble in common organic solvents (chloroform, dichloromethane, dimethyl sulfoxide, methanol and ethanol). The synthesis of complexes **1a** and **1c** and their in vitro antibacterial activity have been previously described; however, the complexes were obtained in a two-step reaction, and the ligand was refluxed in methanol with dibutyl or diphenyl tin(IV) dichloride in the presence of Et_3_N as a base. On the other hand, neither the yields nor the complete NMR, mass spectrometry or UV-Vis characterization was reported [39]. 

The IR spectra (Supplementary Materials, Figures S1–S8) evidenced the formation of organotin(IV) complexes by the absence of the vibrational stretching band ν(OH) as a result of the deprotonation of the phenolic and naphtholic hydroxyls and organotin(IV) complexation; the expected stretching band ν(C=N) corresponding to azomethine was observed from 1599 to 1602 cm^−1^ and exhibits a shift to lower wave numbers in comparison to the free ligand, Δδ = 50 cm^−1^, indicative of the coordination of the azomethine nitrogen to the tin atom [40,41]. Additional evidence of the coordination of the donor atoms N and O from the ligand to the metal was the presence of the ν(Sn-N) and ν(Sn-O) bands found in the ranges 483–489 cm^−1^ and 548–550 cm^−1^, respectively. For complex **1d**, the stretching band ν(Si-C) at 827 cm^−1^ was observed (IR data are provided in Table S1).

The monomeric nature of complexes **1a**–**1d** was revealed by mass spectrometry using the DART (direct analysis in real time) ion source (Figures S9–S12). The spectra showed peaks at *m*/*z* 497,537, 549 and 557, corresponding to the molecular ions [M^+.^] for **1a**–**1d**, respectively. In addition, the expected isotopic peaks where ^119^Sn is the most abundant isotope were observed in all cases. The ^1^H NMR spectra (Figures S13–S16) for all complexes showed the signals for the expected structures. The absence of hydroxylic protons indicates deprotonation and coordination to the tin atom, in agreement with the FTIR observations. The aromatic region exhibits a single signal for the azomethine proton at 10.46–10.56 ppm and shows satellite signals due to the ^3^*J*(^1^H-^117/119^Sn) coupling, which supports Sn-N coordination; the values of the coupling constant were in the range of 49 to 65 Hz. The signals and multiplicities corresponding to the naphtholic and pyridine rings, as well as those corresponding to the organic fragments bonded to tin, were clearly identified; the complete assignment of the individual signals was confirmed by 2D NMR experiments. ^13^C NMR for complexes **1a**–**1d** showed (Figures S17–S20) the iminic carbon in the region of 156.5 to 157.8 ppm. Additional evidence of coordination was found in the aliphatic region; complexes **1a** and **1b** displayed four signals in ranges from 13.6 to 26.9 ppm and from 26.6 to 40.2 for the butyl and cyclohexyl groups bonded to tin. In the case of the **1d** complex, the bis(trimethylsilyl)methyl group showed two single signals for methyl and methylene at 0.0 and 7.1 ppm; meanwhile, in the aromatic region for complex **1c** from 128.0 to 136.4, signals for phenyl groups attached to tin were identified. HSQC and HMBC correlations permitted the identification of all carbon atoms (Figures S21–S28). Satellite signals were observed in most of the complexes, allowing us to calculate the coupling constants *^1^J*(^13^C-^117/119^Sn), *^2^J*(^13^C-^117-/119^Sn), and ^3^*J*(^13^C-^117/119^Sn). From the Holeček equations and the one-bond coupling constants [42,43], C-Sn-C bond angles with values from 120° to 143° were obtained. The ^119^Sn NMR of organotin(IV) complexes was recorded in CDCl_3_; the spectra (Figures S29–S32) showed a single sharp signal in all cases as a result of the formation of discrete species in the solution. It is known that the chemical shift of ^119^Sn NMR is shifted toward lower frequencies as the coordination number increases; however, the type of donor group on tin also has an effect. Dibutyl, bis(trimethylsilyl)methyl and dicyclohexyltin(IV) complexes exhibited less-negative chemical shifts (−191, −154 and −258 ppm) in comparison to diphenyltin(IV) at −332 ppm. In all cases, the values are indicative of a pentacoordinate environment, in accordance with the literature data [39,44,45]. 

**Figure 1:**
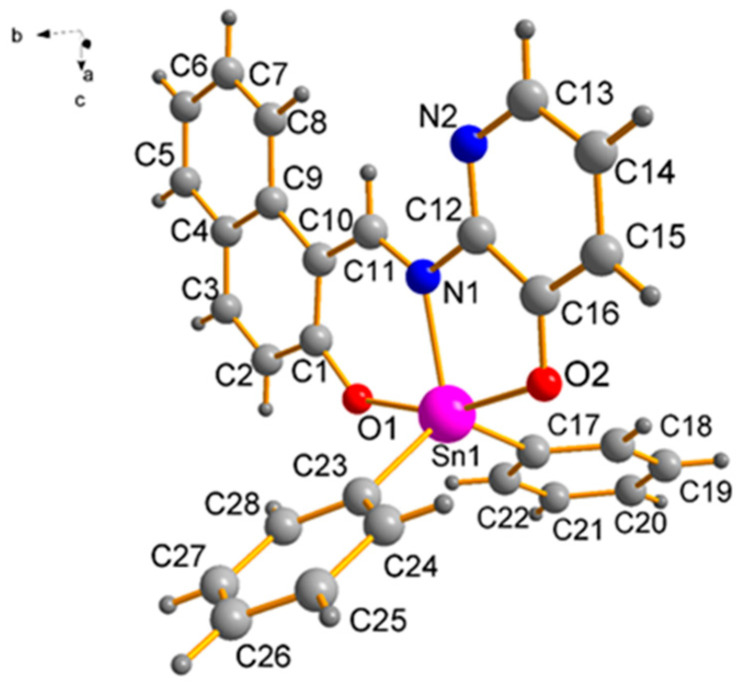
Molecular structure of compound **1c**.

Suitable crystals for single-crystal X-ray diffraction (SCXRD) were obtained only for complex **1c** from dichloromethane. The molecular structure is illustrated in Figure 1. Crystal data are listed in Table 1. Selected bond lengths and angles are summarized in Table 2. Compound **1c** crystallizes in the tetragonal space group I4_1_/a. The tin atom in the mononuclear complex **1c** is coordinated by two carbon atoms of the phenyl groups, two oxygen atoms derived from the tridentate Schiff base ligand and one nitrogen atom of the imine group. The five-coordinate geometry defined by the C_2_NO_2_ donor set is distorted; a measure of distortion away from the ideal trigonal-bipyramidal or square-pyramidal coordination geometries is given by the descriptor *τ* [46], for which these ideal geometries correspond to *τ* = 1.0 and 0.0, respectively. The value *τ* = 0.54 for compound **1c** is indicative of an environment that is intermediate between these extremes. The tendency to prefer a square-pyramidal over a trigonal-bipyramidal geometry is most probably associated with packing effects and intermolecular contacts in the solid state. From the angle values (Table 2), the distortion from the trigonal-bipyramidal or square-pyramidal geometry of compound **1c** can also be observed from the O(1)-Sn(2)-O(2) angle value [161.1(1)°], which is smaller than the ideal 180°. The major distortion in coordination geometry about the tin atom in compound **1c** can be associated with the formation of five-membered (Sn1,O2,C16,C12,N1) and six-membered (Sn1,O1,C1,C10,C11,N1) chelate rings, resulting in tight N1-Sn1-O1 [82.78(8)°] and N1-Sn1-O2 [78.45(9)°] chelate angles, which are shorter than 90°. Thus, the ONO Schiff base behaves as a tridentate coordinating ligand. Five- and six-membered rings result when the N-Sn coordination bond is formed. The annular tension of the formed cycles and the large covalent radius of the tin(IV) atom restrict the planarity of these complexes. The dihedral angles involving these chelate rings are in the range of 0.18(8)° to 6.70(9)°, indicating the non-planarity of the ligand plane around the tin atom (see Table 2).

In compound **1c**, the Sn(1)-O(2) and Sn(1)-O(1) distances at the five- and six-membered rings are both 2.09(2) Å, close to the sum of the covalent radii of Sn-O atoms (2.10 Å), but smaller than that of Van der Waals radii for Sn and O atoms (3.75 Å). These values indicate that these complexes possess strong Sn-O bonds. The Sn(1)-N(1) bond distance is 2.15(2) Å and is equal to the sum of the covalent radii for Sn and N, suggesting a strong tin–nitrogen interaction. This value is comparable to those of analogous diorganotin(IV) complexes containing both five- and six-membered chelate rings, including a set of ONO donor atoms [47,48,49,50]. The Sn(1)-C(17) and Sn(1)-C(23) bond lengths are both 2.12(3) Å, and the bond angle C(17)-Sn(1)-C(23) is 128.5(1)° deviates from the ideal 180° by 51.5°. These values are in accordance with analogous diorganotin(IV) complexes containing phenyl groups attached to the tin atom with a set of ONO donor atoms [51,52]. The C-Sn-C angle decreases with the size increase of alkyl or aryl groups of diorganotin(IV) moieties.

**Figure 2:**
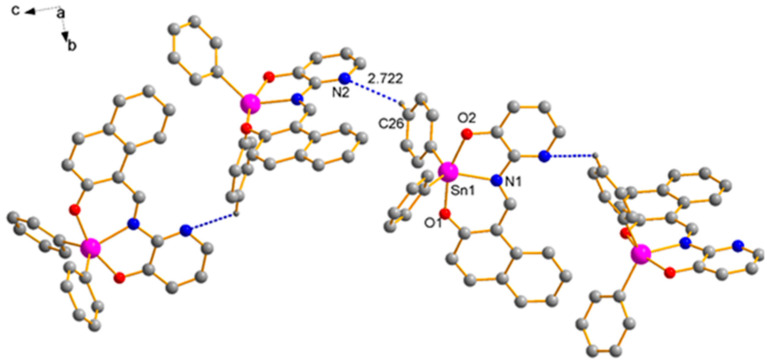
One-dimensional polymeric chain formed by C-H···N (blue dots) interactions in **1c** (symmetry code: i 3/4-x,-1/4+y,-1/4+z).

The structural analysis of **1c** also reveals the presence of C-H···N interactions between the hydrogen atom H26A^i^ of the phenyl group attached to the tin atom and the nitrogen atom of the aromatic ring of the pyridine group, with a bond length of 2.722 Å (symmetry code: i 3/4−x, −1/4+y, −1/4+z), forming a 1D polymeric structure (Figure 2).

**Figure 3:**
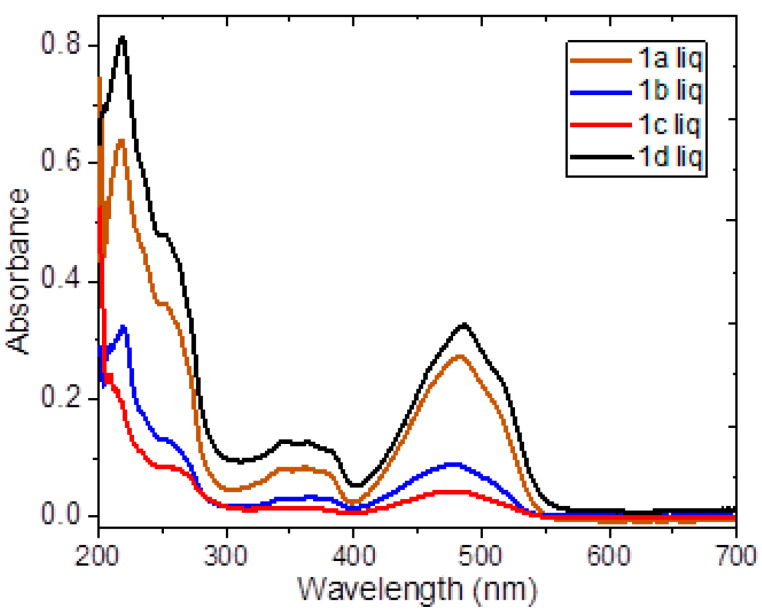
Electronic absorption spectra of **1a**–**1d** recorded in methanol at a concentration of 2.04530 × 10^−5^ M.

The molar conductance was measured in a methanol solution. The values were found in the range of 7.5–35.2 (ohm^−1^cm^2^mol^−1^), indicating the non-electrolytic nature of these organotin(IV) compounds; thus, the donor atoms from the ligand are covalently bonded to the metallic center [53]. The UV-Vis absorption spectra of **1a**–**1d** were measured in methanol solution as well (Figure 3). All complexes showed analogous patterns. The two absorption bands in the regions 214–220 nm (ε_max_ = 30,832–131,423 M^−1^ cm^−1^) and 237–256 nm (ε_max_ = 17,567–602,014 M^−1^ cm^−1^) were assigned to the π-π* electronic transition of the aromatic ring; the bands in the ranges 345–371 nm (ε_max_ = 4034–120,862 M^−1^ cm^−1^) and 481–488 (ε_max_ = 13,274–325,429 M^−1^ cm^−1^) nm were attributed to π-π* (C=N) and the non-bonding electron pair n-π* (C=N) of the iminic nitrogen, as observed for similar organotin–Schiff-base complexes [44,54]. 

**Figure 4:**
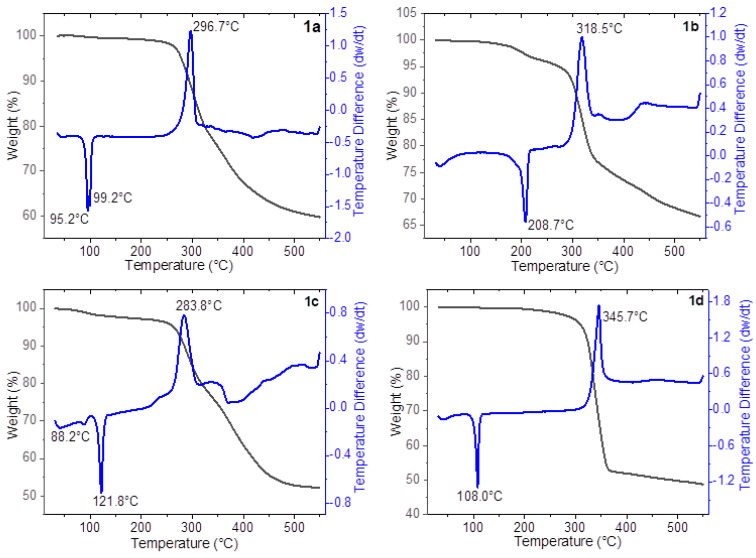
Simultaneous TGA–DSC curves of the thermal decomposition of pentacoordinated organotin(IV) complexes **1a**–**1d**, under flowing dry N_2_ (50 mL·min^−1^) and at a heating rate of 10 °C·min^−1^.

It is important to consider that, for applications in photovoltaics and organic electronics, the optical and electrical behaviors of the compounds in thin films must be tested, and the manufacturing method will depend mainly on the thermal stability of the complexes. Moreover, one must consider whether heating through Joule effects occurs and leads to device failure [55]. The thermal stability of **1a**–**1d** complexes was determined by differential scanning calorimetry (DSC) and thermogravimetric analysis (TGA) over a temperature range from 25 to 550 °C under a nitrogen atmosphere. Figure 4 shows the thermal profiles obtained from the TG/DSC curves for **1a**–**1d** complexes obtained under a nitrogen atmosphere at a heating rate of 10 °C⋅min^−1^ from 25 to 550 °C. Table 3 shows the key temperature points of the TG and DSC curves: the initial temperature decomposition (T_0_), the temperature at the peak maximum (T_max_) of every stage, weight loss due to decomposition and the final decomposition temperature (T_f_). In general terms, the degradation profiles of organotin(IV) compounds **1a**, **1b** and **1d** occur in two stages within a temperature range of 150 to 520 °C, with the maximum rate of weight loss at T_max_ 305, 307 and 332 °C, respectively. In contrast, for complex **1c**, three stages of mass loss are identified, with T_f_ at 451 °C. The thermal stability order is **1c** > **1a** > **1d** > **1b**; complex **1b** has the lowest T_0_ (143 °C) and the highest T_f_ (517 °C) as a consequence of a longer degradation process. The DSC analysis shows endothermic peaks from 95 to 121 °C, attributed to the water of crystallization for **1a**, **1c** and **1d**. [56] In the case of **1b**, the endothermic peak at 208 °C could be associated with coordinated water. The peaks at 296, 318, 283 and 345 °C for **1a**–**1d** correspond to the highest mass loss due to the ligand moiety and organic groups bonded to the tin atom through the mechanism LSnR2-R2SnO-SnO2 [57]. Interestingly, though this stage implies mass loss according to the TGA curves, the DSC curves show a marked exothermic character. The exothermic character of this stage, contrary to the endothermic character expected for the most prominent thermal degradation stage of these complexes, suggests that the evolved energy corresponds to secondary chemical recombination processes at the gas–gas interface, but not to the energy required for its degradation. This behavior shows the complexity of the process and reveals the concurrency of different competitive and sequential phenomena. However, the complexes show adequate thermal stability, which permits their deposition as thin films with the spin-coating technique. The study of these compounds in the solid state will determine their potential for use in photovoltaic devices.

### 2.2. Topographical and Optical Characterization of Hybrid Film

**Figure 5:**
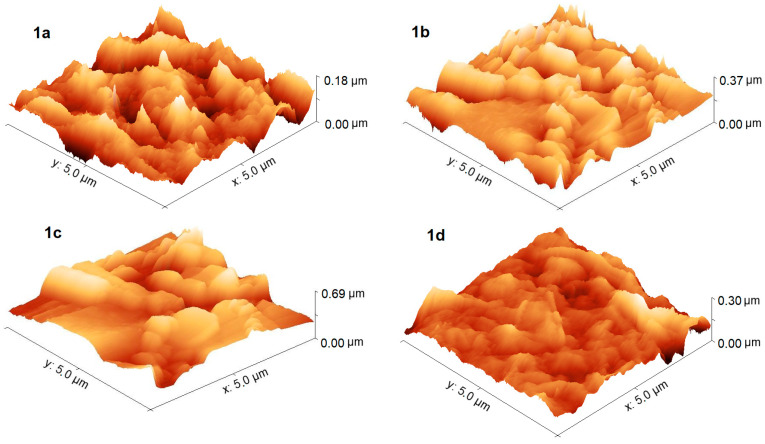
AFM topography of PEDOT:PSS-graphene-organotin(IV) films.

After depositing the PEDOT:PSS-graphene-pentacoordinated organotin(IV) hybrid films by spin coating, AFM was performed in order to determine both the topography and homogeneity of the hybrid films. Film surface roughness is one of the most important parameters for optoelectronic devices, since roughness causes an increase in film resistance and a decrease in operational performance [58,59]. Table 4 shows the root-mean-square (RMS) roughness; the average roughness (R_a_) and AFM images of the surface topography in a 5 × 5 μm area are shown in Figure 5. The highest RMS and Ra roughness occur according to the following trend: **1b** > **1c** > **1d** > **1a**. The different roughnesses and topographies in Figure 5 are related to the type of tin complex used. The complex with the cyclohexyl substituent (**1b**) corresponds to the film with the highest roughness, probably due to poor molecular stacking as a result of the geometry, structure and steric hindrance of the substituent. The roughness of this film is almost twice the roughness of the film with complex **1a**, which has the aliphatic butyl radical. This suggests that the **1a** complex particles are better embedded in the PEDOT:PSS matrix. The above also applies to the film with complex **1d** (phenyl substituent), which has a roughness slightly larger than **1a**. Finally, the film with complex **1c** shows high roughness, which, although smaller than that of **1b**, can affect charge transport in the hybrid film. It seems that the phenyl substituent also affects the incorporation of the complex into the polymeric matrix and its overlap with other organotin complex molecules. Regarding the mechanical properties of the hybrid films, Table 4 shows the unitary deformation (ε), the maximum stress (σ_max_) and the Knoop microhardness (HK), considering a maximum applied force of 990 N. The film with complex **1b** is the one with the highest hardness and the highest mechanical resistance. These mechanical parameters are related to roughness: the highest hardness and mechanical resistance are directly proportional to the roughness and follow the trend **1b** > **1c** > **1d** > **1a**. As for film deformation, the results shown in Table 4 indicate that it is not closely related to roughness; rather, deformation is apparently related to the structure of the organotin(IV) complex.

**Figure 6:**
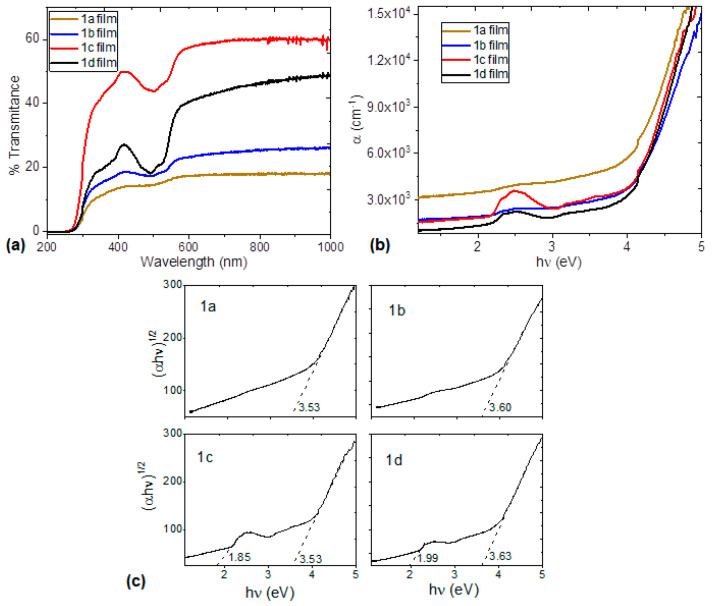
(**a**) Transmittance spectra, (**b**) absorption coefficient spectra and (**c**) Tauc plots of hybrid films.

UV-Vis spectroscopy of the hybrid films was performed to supplement the results obtained in solution. If the use of organotin(IV) complexes in photovoltaic devices is required, it is necessary to understand their solid-state behavior. Regarding the films’ transmittance, one aspect to notice in the spectrum in Figure 6a is the low transmittance of the four films. At a larger wavelength of λ > 580 nm, the films with complexes **1a** and **1b** do not exceed 25% transmittance, film **1d** presents around 50%, and the film with complex **1c**, which is the most transparent, has around 60%. These values are lower than those presented by hybrid films with PEDOT:PSS and graphene from heptacoordinated organotin(IV) complexes [60], although the film with compound **1c** could be used as an electrode in photovoltaic devices. Additionally, in the spectrum, an absorption band is observed in the region between 430 and 570 nm. This band is attributed to the non-bonding electron pair π-π* (C=N) of the iminic nitrogen in the organotin complexes [44,54]. The presence of PEDOT:PSS and graphene slightly shifts this band toward red with respect to the complexes in solution. On the other hand, the absorption coefficient (*α*) for the hybrid films is experimentally obtained from [61]:

α = (1/d) ln 1/T

where d is the thickness of the film (see Table 4), and T is the transmittance; reflectivity (R) is neglected due to its low level for each film (2%), which is caused by the presence of graphene in PEDOT:PSS [35]. Figure 6b shows α as a function of hv for the hybrid films. The presence of PEDOT:PSS and graphene considerably decreases film absorption, and the film with the **1d** complex has the lowest absorption. It is important to consider that α is in the range 10^3^–10^4^ cm^−1^, confirming its potential as a transparent material for solar applications [62].

The absorption edge can be characterized in terms of the absorption edge parameters: (i) the Urbach energy (*E_U_*) and (ii) the energy gap (*E_g_*). The Urbach energy corresponds to the width of the band tail, which is related to localized states within the energy gap, possibly caused by structural defects [61]. The bulk heterojunction formed between the organotin complexes, PEDOT:PSS and graphene, including their interfaces, may be a source of structural defects. Moreover, the behavior of α in the low-energy range follows the exponential law given by Urbach’s expression [61,63].

$$\alpha \propto exp\left(\frac{hv}{E_{U}}\right)$$

The *E_U_* values of hybrid films were calculated from the slope of the exponential edge and are shown in Table 5. As a reference, the value of *E_U_* is zero in a perfect semiconductor [62]. The highest *E_U_* belongs to the film with the **1a** complex, and the lowest *E_U_* belongs to the film with the **1c** complex. From these results, the film with the **1c** complex shows the best semiconducting behavior with the smallest number of defects when compared with the other films. Apparently, the phenyl substituent favors electron delocalization, a better overlap among the complex molecules and a larger integration into the PEDOT:PSS-graphene matrix. While these *E_U_* values are larger than those for some inorganic semiconductor films, such as Bi_2_S_3_ (0.26 eV) and Bi_2−x_Cr_x_S_3_ (0.35 eV) [62], they are nevertheless in the same range as those for inorganic semiconductor films, such as Er-doped ZnO (0.49–0.67 eV) [64]. They are also in the same range as those for hybrid films of the polymer:fullerene blend type, such as polyphenyl-C61-butyric acid methyl ester (0.42–0.48 eV) [61]. Unfortunately, there is not much information available about the E_U_ values for tin-complex thin films in their different coordination states.

*E_g_* is the most relevant parameter in evaluating the potential of hybrid films for use in optoelectronic and photovoltaic devices as active layers. It was calculated through Tauc’s model, which is based on the linear approximation to the energy axis according to the following relation [65,66]:

$$\alpha \propto (hv-E_{g}{)}^{r}\;\mathrm{for}\;\mathrm{the}\;\mathrm{energy}\;\mathrm{range}\;hv>E_{g}$$

where *h* is Planck’s constant, and *r* is a number that characterizes the transition process, with *r* = 2 for indirect transitions in amorphous films. The frequency (ν) is experimentally obtained from:

$$\nu =\frac{c}{\lambda }$$

In this expression, *c* is the speed of light, and *λ* is the wavelength. The dependence of $(\alpha h\nu {)}^{r}$ on hν was plotted, and *E_g_* was evaluated from the *x*-axis intercept at (α*hν*)^1/2^ = 0 for amorphous structures. The values of *E_g_* calculated for the films are shown in Figure 6c and Table 5, and there are important differences among them. Considering that all of the films were deposited under the same conditions and stoichiometric amounts, the changes in their optical behavior can be attributed to the substituent bonded to the tin atom. Additionally, films **1c** and **1d** show two transitions; the first transition is at the onset gap $\left(E_{g}^{onset}\right)$, and the second one corresponds to *E_g_* [65,66]. The electronic transitions from π to π* explain *E_g_* and $E_{g}^{onset}$ as a consequence of several factors, including defects, structural disorder and traps. The two transitions are smaller for the film with complex **1c**, which has a phenyl substituent in its structure. The obtained values are of the same magnitude order as those for heptacoordinated tin(IV) complexes embedded in PEDOT:PSS and graphene ($E_{g}^{onset}$ = 1.13–1.29 eV and *Eg* = 2.83–3.47 eV) [29,30,60]. They are also within the range for inorganic semiconductors. Considering the chemical shifts of ^119^Sn NMR, the phenyl group is the one with the highest donor capacity, and this may be the cause of its lower band gap, in addition to the presence of the highly aromatic electrons of the organotin complex. It seems that **1c** is the one among the four films with the greatest potential for use in optoelectronic and photovoltaic devices due to the fact that its *E_g_* and *E_U_* values are the smallest. Film **1c** is also the one with the greatest transparency.

### 2.3. Device Fabrication and Electrical Characterization

**Figure 7:**
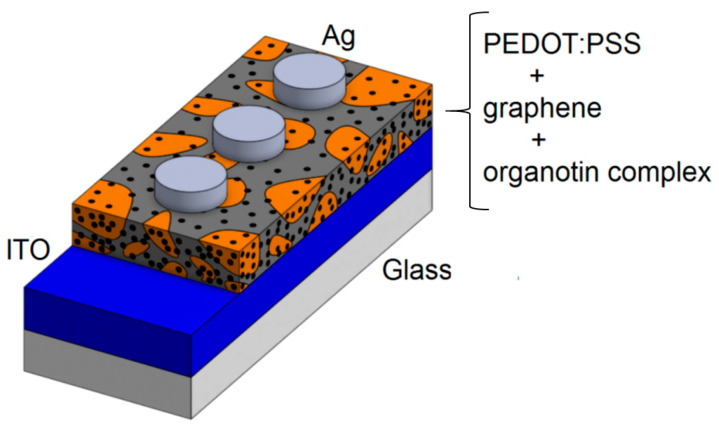
Device schematics.

**Figure 8:**
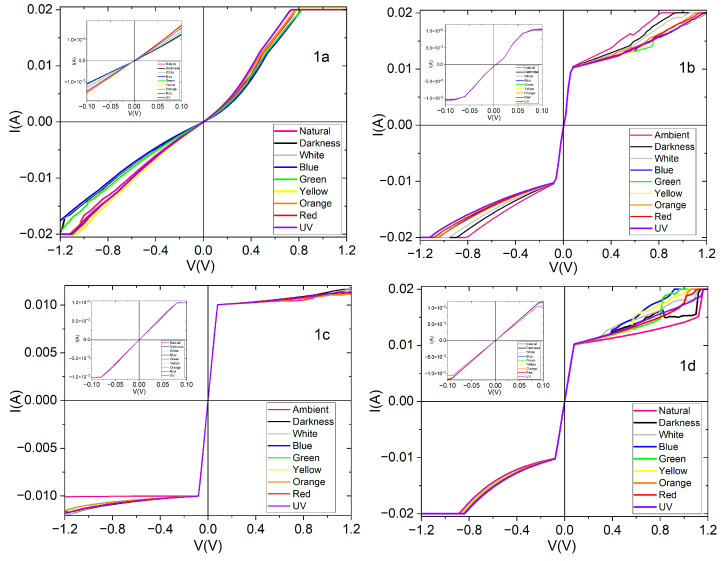
I–V curves from ITO/PEDOT:PSS-graphene-organotin(IV) complex/Ag devices under different lighting conditions.

In order to investigate the electrical properties of the hybrid films, simple devices with only one layer were fabricated, and their current–voltage (I-V) characteristics were measured under different lighting conditions and in darkness. In these devices, the PEDOT:PSS-graphene-organotin(IV) complex hybrid film was deposited over the anode, corresponding to the ITO film. The circuit closes with the silver cathode, which is deposited above the hybrid film (see Figure 7); the electrical behavior is shown in Figure 8. According to their I-V behavior, at low voltages, the devices do not undergo significant modification when subjected to radiation of different wavelengths. Even when enlarging the regions between −0.1 and +0.1 V in the graph, it is observed that there is no effect on device charge transport for the different radiation types. Voltages larger than 0.1 V are required for the ITO-injected holes and Ag-injected electrons to break the hybrid-film barrier, which is probably generated by the complex-PEDOT:PSS-graphene interfaces. In the low-voltage regions, ambipolar behavior can be observed, which changes with increasing voltage, especially in the devices with the **1b** and **1d** films. Another feature that can be observed in the I-V graphs is a maximum transported current of 0.02 A, which is the same for all of the devices, except for the one with the **1c** film, which only carries half of that current. This is an important result, because the **1c** film shows the best optical behavior in terms of parameters such as the band gap and Urbach energy, yet it carries the smallest current, which could be due to the energy difference between the work function of the device electrodes and the hybrid film.

As mentioned earlier, at low voltages, different lighting conditions do not significantly influence the ohmic behavior of the devices. This ohmic behavior can be described by the following equation [67]:

$$J=\frac{p_{0}eµV}{d}$$

where µ is the hole mobility, *p*_0_ is the thermally excited hole concentration, *e* is the electronic charge, *V* is the applied voltage and *d* is the film thickness. The ohmic behavior changes, with a slope change located at 0.44 V in the device with complex **1a** and transitions in the I–V curve located at around 0.08 V in the other devices. These changes in electrical behavior indicate that the device has reached a space-charge-limited current (SCLC) regime, which is described by an exponential trap distribution [67,68]. By raising the voltage to increase the current, it is possible to create a condition in which the carriers do not migrate fast enough and concentrate in a region of the hybrid film PEDOT:PSS-graphene-organotin(IV) complex. Under these conditions, the device enters the SCLC regime, and its behavior is governed by the following equation [68]:

$$J=N_{v}eµ{\left(\frac{\varepsilon_{0}\varepsilon }{eP_{0}kT_{L}}\right)}^{l}\frac{V^{l+1}}{d^{2l+1}}$$

where *ε*_0_ is the vacuum permittivity (8.85 × 10^−14^ CV^−1^cm^−1^), ε is the dielectric constant of the material, *k* is Boltzmann’s constant, *T_L_* is the temperature parameter characterizing the trap distribution and l is the slope of the ohmic zone, which can be obtained by dividing *T_L_* by the local temperature. A value of 3 can be assumed for the dielectric constant of the hybrid films, given that this value corresponds to the commonly accepted average value for organic semiconductors [68]. The electrical parameters *µ* and *p*_0_, evaluated in ordinary daylight, are shown in Table 6. According to previous studies [28], the organotin(IV) complex in such devices has a carrier mobility of approximately 10^−2^–10^−1^ cm^2^/V^.^s. In this work, the obtained values of µ are quite large compared to similar materials and to heptacoordinated organotin complexes in PEDOT:PSS films [28,69]. They are also similar to PEDOT:PSS films doped with graphene [33], which can be attributed to the presence of graphene in the hybrid films. According to Pasha et al. [70], graphene promotes a screening effect between positively charged PEDOT and negatively charged PSS chains, reducing the coulombic interactions between the chains. At the nanometric level, the reduction in coulombic interactions between PEDOT and PSS chains facilitates phase separation. This allows electrical charges to flow more easily through areas with PEDOT, which is the conductive part of the PEDOT:PSS polymer. Additionally, at the microscopic level, graphene increases the π-π conjugation length in the PEDOT:PSS film, so charge transport increases [71]. From the I–V graphs obtained for each device, it is observed that, while graphene increases charge mobility in the hybrid film, it is the organotin complex, and specifically its substituent, that determines the type and form of electrical behavior in the devices. The electrical properties of the four films indicate the potential of pentacoordinated organotin(IV) complexes for use in photovoltaic devices.

## 3. Materials and Methods

All reagents and solvents were obtained from commercial suppliers (Merck KGaA, Darmstadt, Germany) and used without further purification. Cyclohexyl- and bis[(trimethylsilyl)methyl]tin oxides were prepared using the method previously described in the literature [72].

### 3.1. Physical Measurements

Melting points were measured with a Fischer-Johns MEL-TEMP II (Thermo Scientific, Waltham, MA, USA) apparatus and are not corrected. IR spectra were obtained with a Bruker Tensor 27 apparatus (BRUKER, Ettlingen, Germany) by means of the attenuated total reflection (ATR) technique. The NMR spectra of ^1^H, ^13^C and ^119^Sn were acquired using the Bruker Avance III instrument (BRUKER, Rheinstetten, Germany) at 300 MHz, 400 MHz and HD 500 MHz in CDCl_3_. Direct analysis in real time (DART) mass spectra were recorded on a JEOL-JMS-T100LC instrument (JEOL, Tokyo, Japan). The X-ray diffraction study was performed with a BRUKER SMART APEX CCD diffractometer (BRUKER, Karlsruhe, Germany) at a wavelength λ_(Mo-Kα)_ = 0.71073 Ǻ (graphite monochromator) and temperature T = 298 K for complex **1c**. The structure was solved using direct methods in the SHELXS program. All non-hydrogen atoms were anisotropically refined using a full-matrix, least-squares technique, and all hydrogen atoms were placed in idealized positions using the riding hydrogen model approximation. Structural solutions and refinements were performed using SHELXTL [73]. The molecular structure and packing were drawn with DIAMOND [74]. Molar conductivity measurements were recorded with a Hanna 6484 system using anhydrous methanol as a solvent. The UV-Vis spectra were recorded using a Cary 50 Varian instrument with anhydrous methanol as a solvent at 2.04530 × 10^−5^ M. The study of the thermal degradation was carried out using simultaneous TGA-DSC (Simultaneous Thermal Analyzer STA 449 F3 Jupiter, NETZSCH-Gerätebau GmbH - Selb, Germany). In each trial, a 4 mg constant-mass sample was placed in a 25 μL aluminum crucible and then heated from room temperature to 550 °C under a nitrogen atmosphere and using a total flow rate of 50 mL min^−1^.

### 3.2. General Procedure for the Synthesis of Diorganotin(IV) Compounds ***1a**–**d***

2-Hydroxy-1-naphthaldehyde and the corresponding diorganotin oxide were added in a stoichiometric ratio (1:1:1) to a solution of 2-amino-3-hydroxypyridine. The reaction mixture was refluxed for 30 h using a Dean–Stark trap in the case of complexes **1a** and **1d,** while the corresponding time for complexes **1b** and **1c** (containing the phenyl and cyclohexyl moieties) was 48 h. Afterward, the solvent was eliminated under reduced pressure to produce the desired complexes. All complexes were purified by methanol recrystallization. 

### 3.3. 2,2-Dibutyl-6-aza-1,3-dioxa-2-stannanaphtho[1,2-h]pyrido[3,2-d]cyclononene *(**1a**)*

Compound **1a** was prepared from 0.157 g (0.907 mmol) of 2-hydroxy-1-naphthaldehyde, 0.101 g (0.907 mmol) of 2-amino-3-hydroxypyridine and 0.2264 g (0.907 mmol) of dibutyltin oxide, producing 0.376 g (84%) of a brown solid; m.p. 87–89 °C. (dec); molar conductance**,** Λ_M_ (1 × 10^−3^ M, methanol): 8.7 μS cm^−1^; UV-Vis [methanol, λ_máx_/nm (ε/M^−1^ cm^−1^)]: 219 (30,832), 253 (17,567) π → π* (aromatic), 363 (4034) π → π* (C=N), 483 (13,274) n → π* (C=N); **IR** (ATR) cm^−1^: 3043 ν(C-H), 2948 ν_as_(C-H), 2920 ν_as_(C-H), 2865 ν_sim_(C-H), 2846 ν_sim_(C-H), 1601 ν(C=O), 606 ν(Sn-C), 548 ν(Sn-O), 483 ν(Sn-N); ^1^H NMR (300.18 MHz, CDCl_3_) δ: 0.85 (6H, t, *J* = 7.51 Hz, H-δ), 1.34 (4H, Sext, *J* = 7.51, H-γ), 1.50–1.55 (4H, m, H-α), 1.61–1.72 (4H, m, H-β), 6.89 (1H, d, *J* = 9.32 Hz, H-2), 7.07–7.08 (2H, H-14, H-15), 7.33 (1H, ddd, *J* = 0.90, 7.21, 7.21 Hz, H-6), 7.56 (1H, ddd, *J* = 1.50, 7.21, 7.21 Hz, H-7), 7.67 (1H, dd, *J* = 0.90, 7.81 Hz, H-5), 7.78 (1H, dd, *J* = 2.40, 3.91 Hz, H-13), 7.81 (1H, d, *J* = 9.32 Hz, H-3), 8.26 (1H, d, *J* = 8.41 Hz, H-8), 10.48 (1H, s, ^3^*J*(^1^H-^117/119^Sn) = 52 Hz, H-11); ^13^C NMR (100.62 MHz, CDCl_3_) δ: 174.2 (C-1), 157.7 (C-11), 153.8 (C-16), 145.2 (C-12), 139.7 (C-3), 135.2 (C-13), 134.9 (C-9), 129.4 (C-5), 128.7 (C-7), 127.1 (C-4), 124.8 (C-2), 124.5 (C-14), 124.3 (C-15), 123.6 (C-6), 119.5 (C-8), 108.8 (C-10), 26.9 (^2^*J*(^13^C-^117/119^Sn) = 40 Hz, C-β), 26.6 (C-γ), 22.4 (C-α), 13.6 (C-δ); ^119^Sn NMR (112.04 MHz, CDCl_3_) δ: −191; **MS**: (DART^+^) [*m*/*z*] (%): [(M^+^ + 1), 497] (33). HR-MS (DART^+^) *m*/*z:* 497.12151 (calc. for^12^C_24_^1^H_29_^14^N_2_^16^O_2_^120^Sn), observed: 497.12384.

### 3.4. 2,2-Dicyclohexyl-6-aza-1,3-dioxa-2-stannanaphtho[1,2-h]pyrido[3,2-d]cyclononene *(**1b**)*

Compound **1b** was prepared from 0.236 g (1.365 mmol) of 2-hydroxy-1-naphthaldehyde, 0.151 g (1.365 mmol) of 2-amino-3-hydroxypyridine and 0.410 g (1.365 mmol) of dicyclohexyltin(IV) oxide, producing 0.409 g (55%) of a brown solid; m.p. 69–71 °C; molar conductance, Λ_M_ (1 × 10^−3^ M, methanol): 7.9 μS cm^−1^; UV-Vis [methanol, λ_máx_/nm (ε/M^−1^ cm^−1^)]: 214 (104,238), 256 (40,336) π → π* (aromatic), 347 (7285) π → π* (C=N), 485 (20,730) n → π* (C=N); IR (ATR) cm^−1^: 2916 ν_as_(C-H), 2845 ν_sim_(C-H), 1599 ν(C=O), 601 ν(Sn-C), 548 ν(Sn-O), 488 ν(Sn-N); ^1^H NMR (500.17 MHz, CDCl_3_) δ: 1.50–2.17 (22H, m, H-α, H-β, H-γ, H-δ), 6.93 (1H, d, *J* = 9.20 Hz, H-2), 7.06 (1H, dd, *J* = 4.50, 8.00 Hz, H-15), 7.11 (1H, dd, *J* = 1.50, 8.00 Hz, H-14), 7.32 (1H, ddd, *J* = 0.50, 7.00 Hz, H-6), 7.55 (1H, ddd, *J* = 1.00, 7.00 Hz, H-7), 7.67 (1H, dd, *J* = 1.00, 8.00 Hz, H-5), 7.76 (1H, dd, *J* = 1.50, 4.00 Hz, H-13), 7.81 (1H, d, *J* = 7.95 Hz, H-3), 8.27 (1H, d, *J* = 8.50 Hz, H-8), 10.56 (1H, s, ^3^*J*(^1^H-^117/119^Sn) = 49 Hz, H-11); ^13^C NMR (100.62 MHz, CDCl_3_) δ: 174.7 (C-1), 157.6 (C-11), 154.4 (C-16), 145.5 (C-12), 139.6 (C-3), 135.0 (C-9), 134.9 (C-13), 129.3 (C-5), 128.6 (C-7), 127.1 (C-4), 125.0 (C-2), 124.4 (C-14), 124.2 (C-15), 123.5 (C-6), 119.5 (C-8), 108.8 (C-10), 40.2 (^1^*J*(^13^C-^119/117^Sn) = 600, 574 Hz, C-α), 30.9 (^2^*J*(^13^C-^119^Sn) = 25 Hz, C-β), 28.6 (^3^*J*(^13^C-^119/117^Sn) = 94, 81 Hz C-γ), 26.6 (C-δ); ^119^Sn NMR (149.18 MHz, CDCl_3_) δ: −258; MS: (DART^+^) [*m*/*z*] (%): [(M^+^ + 1), 549] (17); HR-MS (DART^+^) *m*/*z:* 549.15640 (calc. for^12^C_28_^1^H_33_^14^N_2_^16^O_2_^120^Sn), observed: 549.15405.

### 3.5. 2,2-Diphenyl-6-aza-1,3-dioxa-2-stannanaphtho[1,2-h]pyrido[3,2-d]cyclononene *(**1c**)*

Compound **1c** was prepared from 0.157 g (0.910 mmol) of 2-hydroxy-1-naphthaldehyde, 0.101 g (0.910 mmol) of 2-amino-3-hydroxypyridine and 0.2631 g (0.910 mmol) of diphenyltin(IV) oxide, producing 0.275 g (57%) of an orange solid; m.p. 148–150 °C; molar conductance, Λ_M_ (1 × 10^−3^ M, methanol): 10.6 μS cm^−1^; UV-Vis [methanol, λ_máx_/nm (ε/M^−1^ cm^−1^)]: 220 (77,225), 255 (31,242) π → π* (aromatic), 371 (7529) π → π* (C=N), 481 (21,488) n → π* (C=N); **IR** (ATR) cm^−1^: 3044 ν(C-H), 2948 ν_as_(C-H), 2921 ν_as_(C-H), 2866 ν_sim_(C-H), 2847 ν_sim_(C-H), 1601 ν(C=O), 607 ν(Sn-C), 550 ν(Sn-O), 485 ν(Sn-N); ^1^H NMR (400.13 MHz, CDCl_3_) δ: 7.11 (1H, dd, *J* = 4.40, 8.00 Hz, H-14), 7.19 (1H, d, *J* = 9.20 Hz, H-2), 7.28 (1H, dd, *J* = 0.40, 6.80 Hz, H-6), 7.32 (1H, d, *J* = 1.29, 8.00 Hz, H-15), 7.37–7.42 (6H, m, H-*m*, H-*p*), 7.50 (1H, ddd, *J* = 1.20, 7.20, 7.20 Hz, H-7), 7.64 (1H, dd, *J* = 1.20, 8.00 Hz, H-5), 7.80 (1H, dd, *J* = 1.20, 4.40 Hz, H-13), 7.87 (1H, d, *J* = 9.20 Hz, H-3), 7.90–7.96 (4H, m, H-*o*), 8.20 (1H, d, *J* = 8.40 Hz, H-8), 10.46 (1H, s, ^3^*J*(^1^H-^117/119^Sn) = 65 Hz, H-11); ^13^C NMR (100.62 MHz, CDCl_3_) δ: 174.1 (C-1), 157.8 (C-11), 153.3 (C-16), 144.7 (C-12), 140.2 (C-3), 139.6 (^1^*J*(^13^C-^119^Sn) = 1062 Hz, C-*i*), 136.4 (^2^*J*(^13^C-^117/119^Sn) = 60.4, C-*o*), 135.6 (C-13), 134.7 (C-9), 130.5 (C-*m*), 129.5 (C-5), 128.9 (^4^*J*(^13^C-^117/119^Sn) = 90.5, C-*p*, C-7), 127.4 (C-4), 125.1 (C-15), 124.8 (C-2), 124.5 (C-14), 124.0 (C-6), 119.6 (C-8), 109.2 (C-10); ^119^Sn NMR (112.04 MHz, CDCl_3_) δ: −332; MS: (DART^+^) [*m*/*z*] (%): [(M^+^ + 1), 537] (85). HR-MS (DART^+^) *m*/*z:* 537.06250 (calc. for ^12^C_28_^1^H_21_^14^N_2_^16^O_2_^120^Sn), observed: 537.05877.

### 3.6. 2,2-Bis(trimethylsilyl)methyl-6-aza-1,3-dioxa-2-stannanaphtho[1,2-h]pyrido[3,2-d]cyclononene *(**1d**)*

Compound **1d** was prepared from 0.235 g (1.364 mmol) of 2-hydroxy-1-naphthaldehyde, 0.1503 g (1.364 mmol) of 2-amino-3-hydroxypyridine and 0.4210 g (1.364 mmol) of bis[(trimethylsilyl)methyl]tin(IV) oxide, producing 0.7156 g (95%) of a red solid; m.p. 88–90 °C; molar conductivity, Λ_M_ (1 × 10^−3^ M, methanol): 23.6 μS cm^−1^; UV-Vis [methanol, λ_máx_/nm (ε/M^−1^ cm^−1^)]: 219 (131,423), 256 (75,897) π → π* (aromatic), 348 (20,665) π → π* (C=N), 488 (52,559) n → π* (C=N); **IR** (ATR) cm^−1^: 3046 ν(C-H), 2948 ν_as_ (C-H), 2890 ν_sim_(C-H), 1602 ν(C=O), 603 ν(Sn-C), 549 ν(Sn-O), 488 ν(Sn-N); ^1^H NMR (400.13 MHz, CDCl_3_) δ: 0.00 (18H, H-β), 0.48 ( (4H, d, *J* = 4.00, H-α), 6.85 (1H, d, *J* = 9.20 Hz, H-2), 7.05–7.08 (2H, m, H-14, H-15), 7.34 (1H, ddd, *J* = 0.80, 7.60, 7.60 Hz, H-6), 7.57 (1H, ddd, *J* = 1.20, 7.20, 7.20 Hz, H-7), 7.68 (1H, dd, *J* = 1.20, 8.00 Hz, H-5), 7.78 (1H, dd, *J* = 2.20, 3.80 Hz, H-13), 7.82 (1H, d, *J* = 9.20 Hz, H-3), 8.28 (1H, d, *J* = 8.40 Hz, H-8), 10.51 (1H, s, ^3^*J*(^1^H-^117/119^Sn) = 56 Hz, H-11); ^13^C NMR (125.78 MHz, CDCl_3_) δ: 172.4 (C-1), 156.5 (C-11), 151.9 (C-16), 143.7 (C-12), 138.8 (C-3), 134.2 (C-13), 133.7 (C-9), 128.3 (C-5), 127.6 (C-7), 126.0 (C-4), 123.8 (C-2), 123.5 (C-14), 123.2 (C-15), 122.6 (C-6), 118.4 (C-8), 107.6 (C-10), 7.1 (^1^*J*(^13^C-^117/119^Sn) = 519, C-α), 0.0 (C-β); ^119^Sn NMR (149.18 MHz, CDCl_3_) δ: −154; MS: (DART^+^) [*m*/*z*] (%): [(M^+^ + 1), 557] (16). HR-MS (DART^+^) *m*/*z:* 557.11025 (calc. for ^12^C_24_^1^H_33_^14^N_2_^16^O_2_^120^Sn), observed: 557.10326.

### 3.7. Hybrid Film Fabrication and Characterization

The hybrid films were deposited by the spin-coating technique, and Smart Coater 200 equipment was used. The dispersion used for the manufacture of the films consisted of 6 mL of a graphene-PEDOT:PSS hybrid dispersion in dimethylformamide. Subsequently, a saturated dispersion was generated with the pentacoordinated organotin(IV) complex. The graphene-PEDOT:PSS-organotin(IV) complex mixture was dispersed with the G560 shaker of Scientific Industries Vortex-Genie. The dispersion was later deposited on the substrate, and the equipment was operated at a constant angular speed of 900 rpm for 20 s and then at an accelerated speed of 300 rpm/s and dried at 80 °C for 3 min. Thin films were deposited on glass, silicon wafers (c-Si) and indium tin oxide (In_2_O_3_·(SnO_2_)_x_)-coated glass (glass-ITO) substrates. Previously, the glass and glass-ITO substrates were sequentially washed in an ultrasonic bath with dichloromethane, methanol and acetone. The silicon substrate was washed with a *p* solution (10 mL HF, 15 mL HNO_3_ and 300 mL H_2_O) to remove surface oxides. In order to obtain the quinoid form in the PEDOT:PSS polymer, the films were post-treated by exposure to isopropanol (IPA) vapor while heated at 40 °C for 10 min. For the hybrid films on Corning glass, the UV-Vis spectra were obtained in the 200–1100 nm wavelength range with a UV-Vis 300 Unicam spectrophotometer. Topographic and mechanical characteristics were investigated with an atomic force microscope (AFM) using a Ntegra platform. Finally, the devices were fabricated by using ITO as an anode and silver as a cathode: glass/ITO/organotin(IV) complex/Ag. For this evaluation, a programmable voltage source, a sensing station with lighting and a temperature-controller circuit from Next Robotix and an auto-ranging Keithley 4200-SCS-PK1 pico-ammeter were employed. 

## 4. Conclusions

The synthesis of four pentacoordinated organotin(IV) complexes prepared by one-pot synthesis through the reaction of 2-hydroxy-1-naphthaldehyde, 2-amino-3-hydroxypyridine and organotin oxides was carried out. The organotin(IV) complexes were used to fabricate hybrid films with PEDOT:PSS and graphene, and the film with complex **1b** (cyclohexyl substituent) had the best mechanical (σ_max_ = 1.69 × 10^7^ Pa, HK = 0.061) properties; however, the film with complex **1d** ((trimethylsilyl)methyl substituent) had the best ohmic behavior, and the film with complex **1c** (diphenyl substituent) had the smallest optical band gap ($E_{g}^{onset}=1.85$ and $E_{g}^{optical}=3.53eV$) and an Urbach energy of 0.48 eV. The organotin(IV) complex devices had a maximum electrical current of 0.02 A and carrier mobility of 10^−2^–10^−1^ cm^2^/V^.^s. Graphene increases charge mobility in the hybrid film, and the substituent in the organotin complex determines the type and form of electrical behavior in the devices. The substituent in the organotin(IV) complexes also determines the mechanical and optical behaviors of the hybrid films.

## Figures and Tables

**Table 1:** Crystallographic and structure refinement data for compound **1c**.

Identification Code	1c
Empirical formula	C_28_H_20_N_2_O_2_Sn
Formula weight	535.15
Temperature/K	298(2)
Crystal system	tetragonal
Space group	I4_1_/a
a/Å	17.2213(5)
b/Å	17.2230(5)
c/Å	31.2313(9)
α/°	90
β/°	90
γ/°	90
Volume/Å	90
Z	16
ρ cal g/cm^3^	1.535
μ/mm^−1^	1.131
F(000)	4288
Crystal size/mm^3^	0.37 × 0.37 × 0.23
Radiation	MoKα (λ = 0.71073)
2Ɵ range for data collection/°	2.286 to 25.383
Reflections collected	38,660
Independent reflections	4254[R_int_ = 0.0719]
Data/restraints/parameters	4254/0/298
Goodness-of-fit on F^2^	1.124
Final R indexes [I ≥ 2σ(I)]	R_1_ = 0.0318, wR_2_ = 0.0672
Final R indexes [all data]	R_1_ = 0.0405, wR_2_ = 0.0729

**Table 2:** Selected bond distances (Å) and angles (°) for compound **1c**.

Bond Distances (Å)			
Sn1-N1	2.152(2)	Sn1-C23	2.119(3)
Sn1-O1	2.087(2)	N1-C11	1.308(3)
Sn1-O2	2.089(2)	N1-C12	1.420(4)
Sn1-C17	2.118(3)	N2-C12	1.319(4)
**Bond Angles (°)**			
O1-Sn1-N1	82.8(1)	O2-Sn1-C17	95.8(1)
O1-Sn1-O2	161.1(1)	O2-Sn1-C23	95.2(1)
O1-Sn1-C17	92.1(1)	N1-Sn1-C17	120.2 (1)
O1-Sn1-C23	93.3(1)	N1-Sn1-C23	111.3(1)
O2-Sn1-N1	78.5(5)	C17-Sn1-C23	128.46(11)
**Torsion Angles (°)**			
Sn(1)-N(1)-C(12)-C(16)	1.99	Sn(1)-O(1)-C(1)-C(10)	2.30
Sn(1)-O(2)-C(16)-C(12)	−6.70	C(10)-C(11)-N(1)-C(12)	177.69
Sn(1)-N(1)-C(11)-C(10)	0.18		

**Table 3:** Thermal data (°C) of pentacoordinated organotin(IV) complexes **1a**–**1d**.

Complex	T_0_	T_max_	T_f_
**1a**	253.98	305.23	442.33
**1b**	143.83	307.99	517.77
**1c**	258.87	299.72	451.98
**1d**	175.79	332.93	368.89

**Table 4:** Roughness, dimensions and mechanical parameters of hybrid films.

Complex	RMS (nm)	R_a_ (nm)	Thickness (μm)	Area (m^2^)	Unitary Deformation	σ_max_ (Pa)	HK
**1a**	28.67	22.98	5.3	7.89 × 10^−4^	0.877	9.90 × 10^6^	0.035
**1b**	48.16	36.55	6.2	6.99 × 10^−4^	0.929	1.69 × 10^7^	0.061
**1c**	37.45	28.75	5.8	7.58 × 10^−4^	0.909	1.1 × 10^7^	0.040
**1d**	30.63	23.46	5.1	6.93 × 10^−4^	0.949	1.02 × 10^7^	0.036

**Table 5:** Energy gap and Urbach energy.

Optical Parameter	1a	1b	1c	1d
*E_g_* (eV)	3.53	3.60	3.53	3.63
$E_{g}^{onset}$ (eV)	-	-	1.85	1.99
*E_U_* (eV)	0.71	0.66	0.48	0.59

**Table 6:** Electrical parameters under natural illumination for ITO/PEDOT:PSS-graphene-organotin(IV) complex/Ag devices under daylight conditions.

Electrical Parameter	1a	1b	1c	1d
μ	4.34 × 10^−1^ cm^2^/Vs	3.63 cm^2^/Vs	2.62 × 10^−2^ cm^2^/Vs	1.95 cm^2^/Vs
*p*_0_	4.06 × 10^18^ m^−3^	2.96 × 10^18^ m^−3^	3.93 × 10^18^ m^−3^	4.38 × 10^18^ m^−3^

## Data Availability

Not applicable.

## Supplementary Materials

The following supporting information can be downloaded at https://www.mdpi.com/article/10.3390/ijms24065255/s1.
