# Supplementary material for: Pentacoordinated Organotin(IV) Complexes as an Alternative in the Design of Highly Efficient Optoelectronic and Photovoltaic Devices: Synthesis and Photophysical Characterization
Source: Int J Mol Sci. 2023 Mar 9;24(6):5255. doi: 10.3390/ijms24065255 (PMC10049675; doi:10.3390/ijms24065255)
Supplement: Supplementary file 1 [file ijms-24-05255-s001.zip › ijms-2205345-supplementary.pdf]

Supplementary Materials

Pentacoordinated Organotin(IV) Complexes as an Alternative in the Design of Highly Efficient Optoelectronic and Photovoltaic Devices: Synthesis and Photophysical Characterization

María Elena Sánchez Vergara [1,*], Elizabeth Gómez [2,*], Emiliano Toledo Dircio [1], José Ramón Álvarez-Bada [1], Samuel Cuenca Pérez [2], José Miguel Galván Hidalgo [2], Arturo González Hernández [2] and Simón Hernández Ortega [2]

[1] Facultad de Ingeniería, Universidad Anáhuac México, Avenida Universidad Anáhuac 46, Col. Lomas Anáhuac, Huixquilucan, Estado de México 52786, México.
[2] Instituto de Química, Universidad Nacional Autónoma de México, Circuito Exterior s/n. C.U., Alcaldía Coyoacán, 04510. Ciudad de México, México.

Table S1. Characteristic IR stretching frequencies of compounds 1a-1d

Compound	ν(C=N)	ν(Sn-C)	ν(Sn-O)	ν(Sn-N)
**1a**	1601	606	548	483
**1b**	1601	607	550	485
**1c**	1599	601	548	488
**1d**	1602	603	549	488

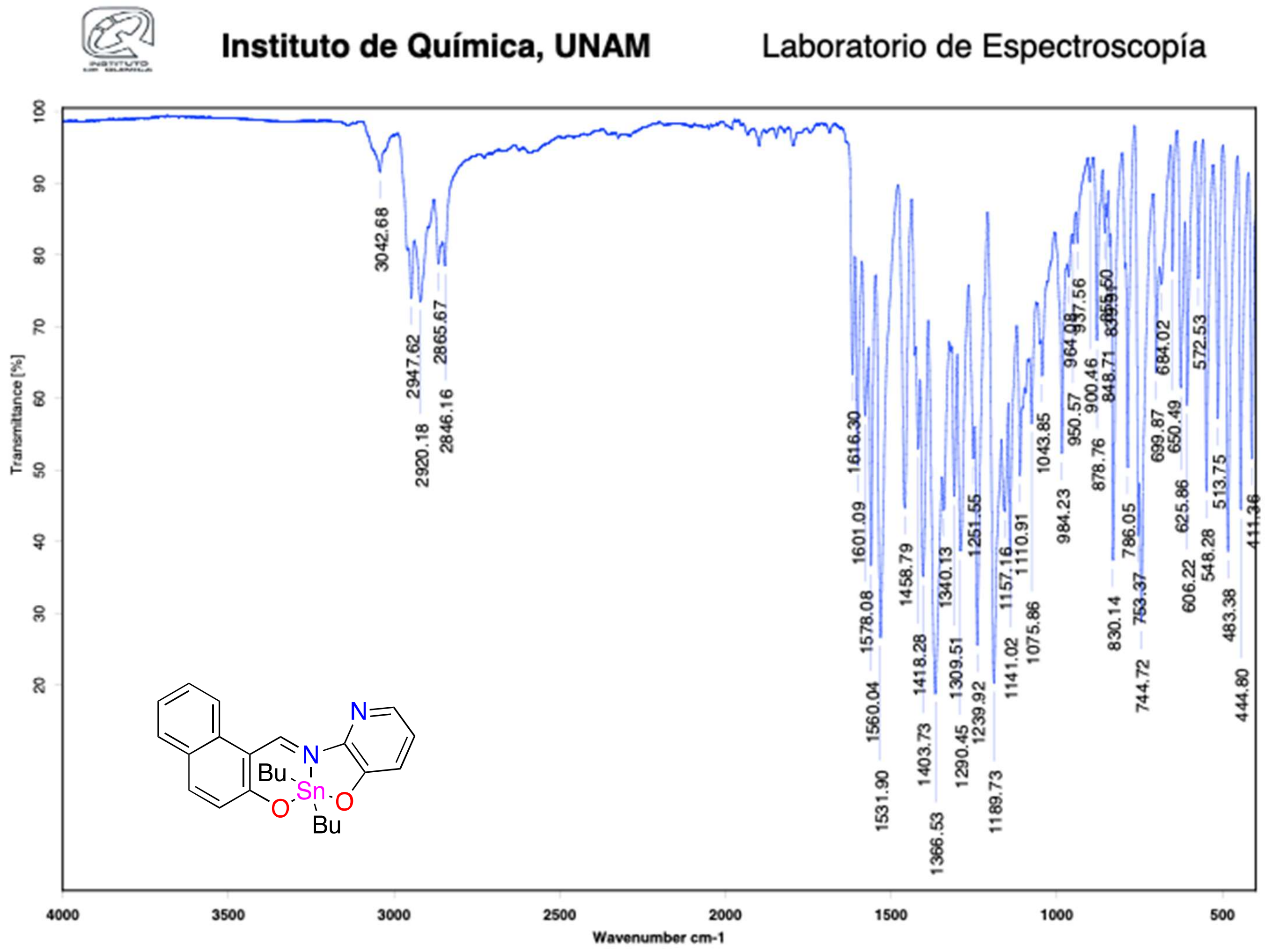

Figure S1. ATR-IR of complex **1a**

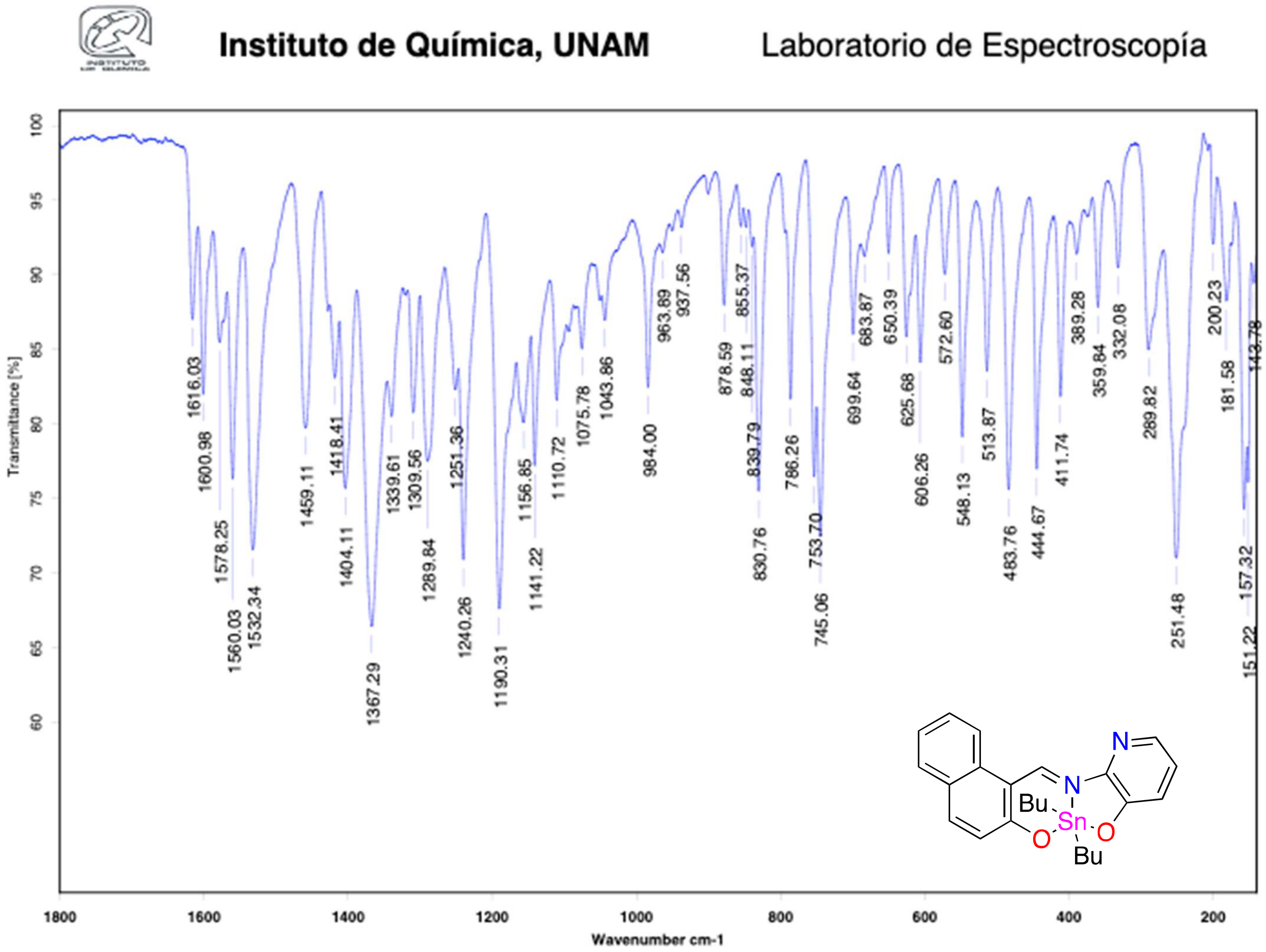

Figure S2. Far-IR of complex **1a**

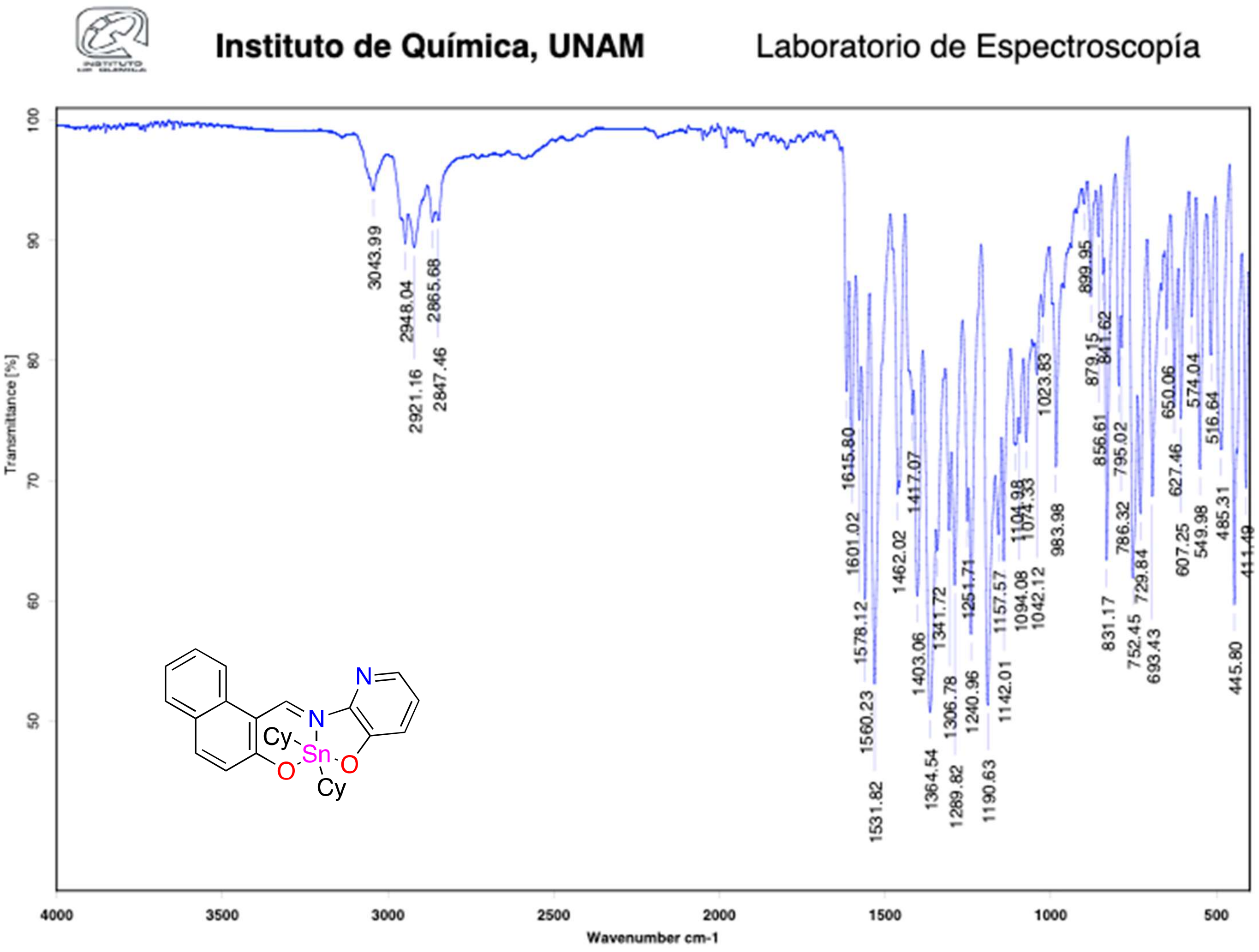

Figure S3. ATR-IR of complex **1b**

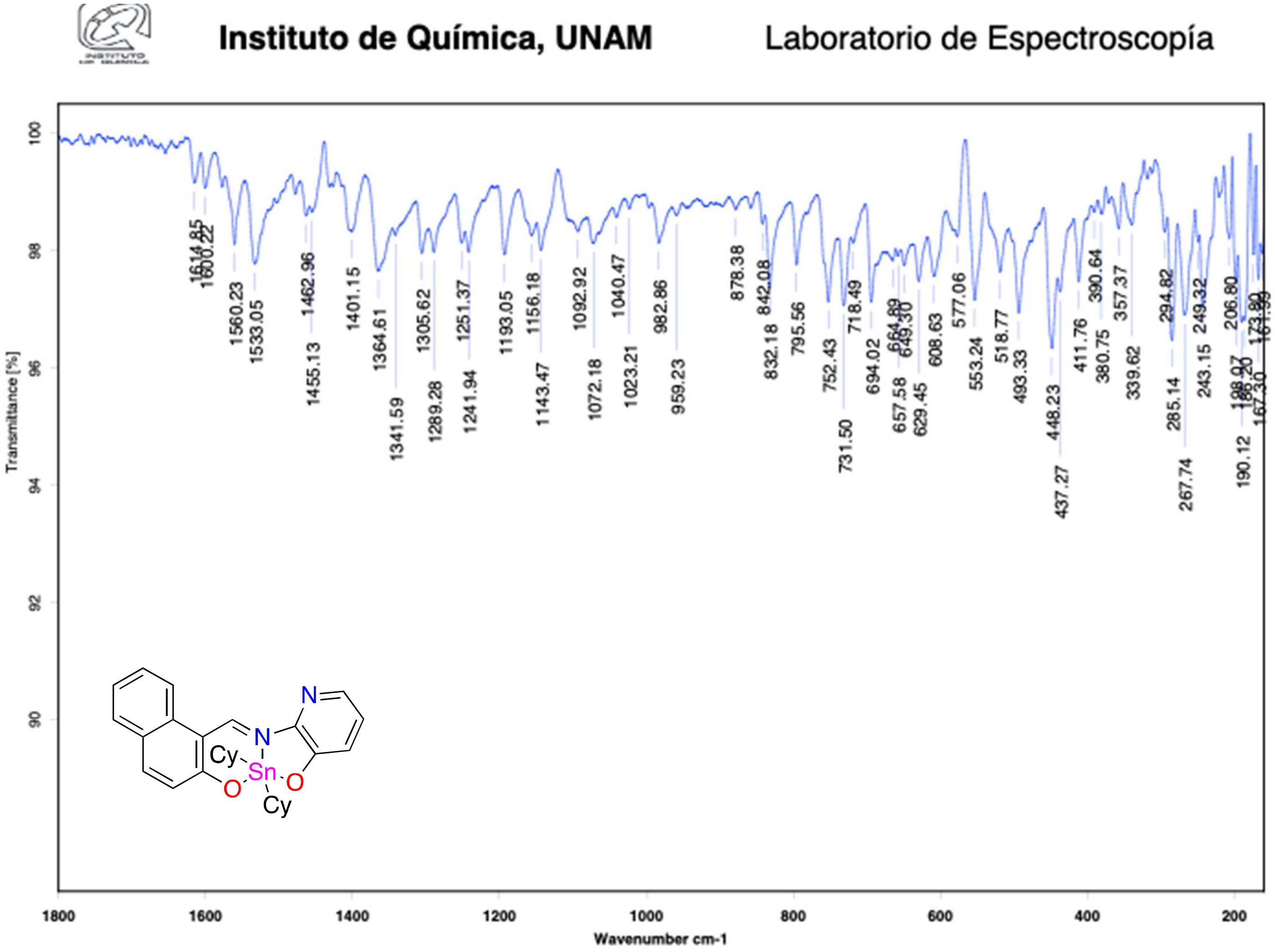

Figure S4. Far-IR of complex **1b**

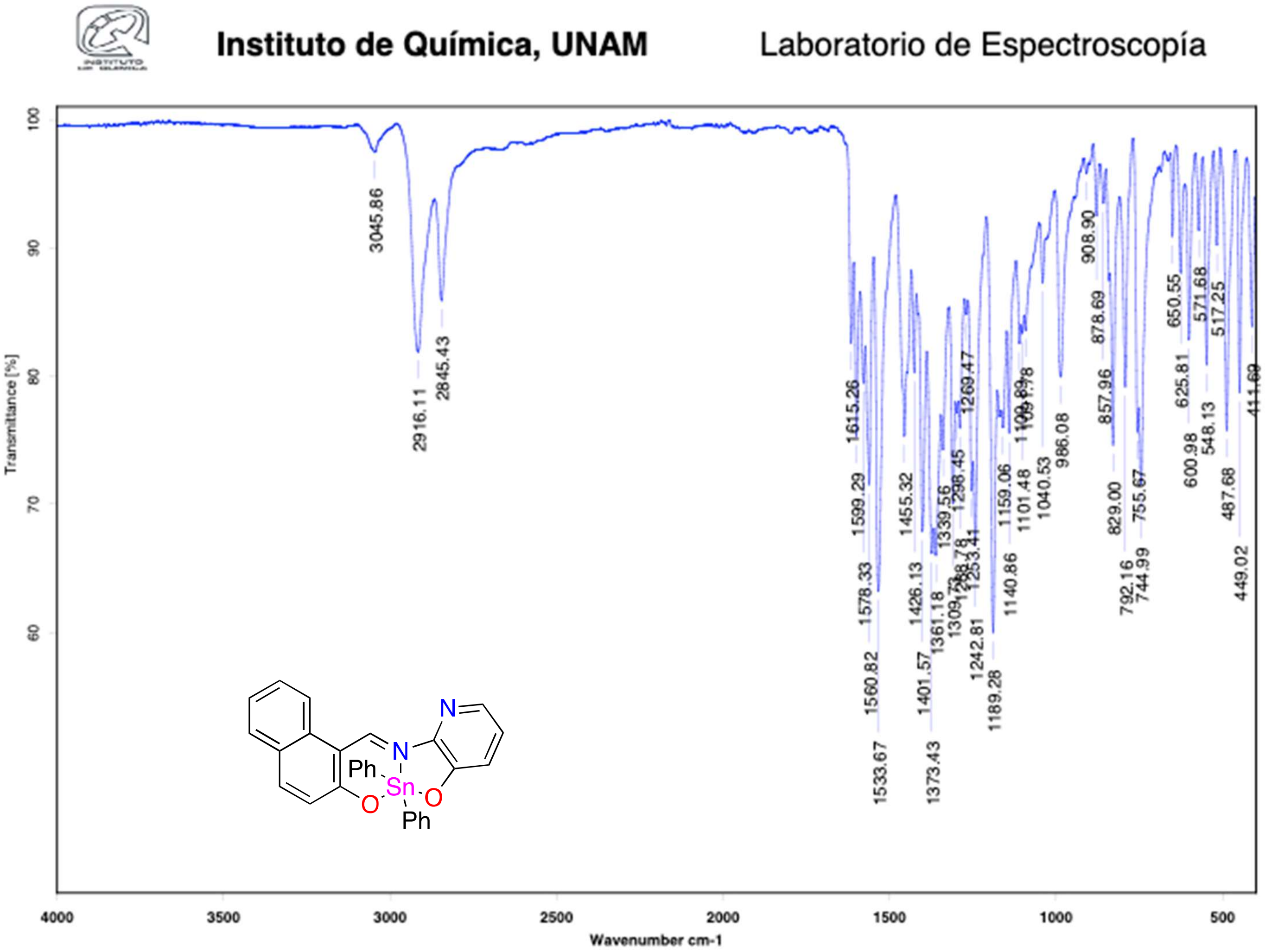

Figure S5. ATR-IR of complex **1c**

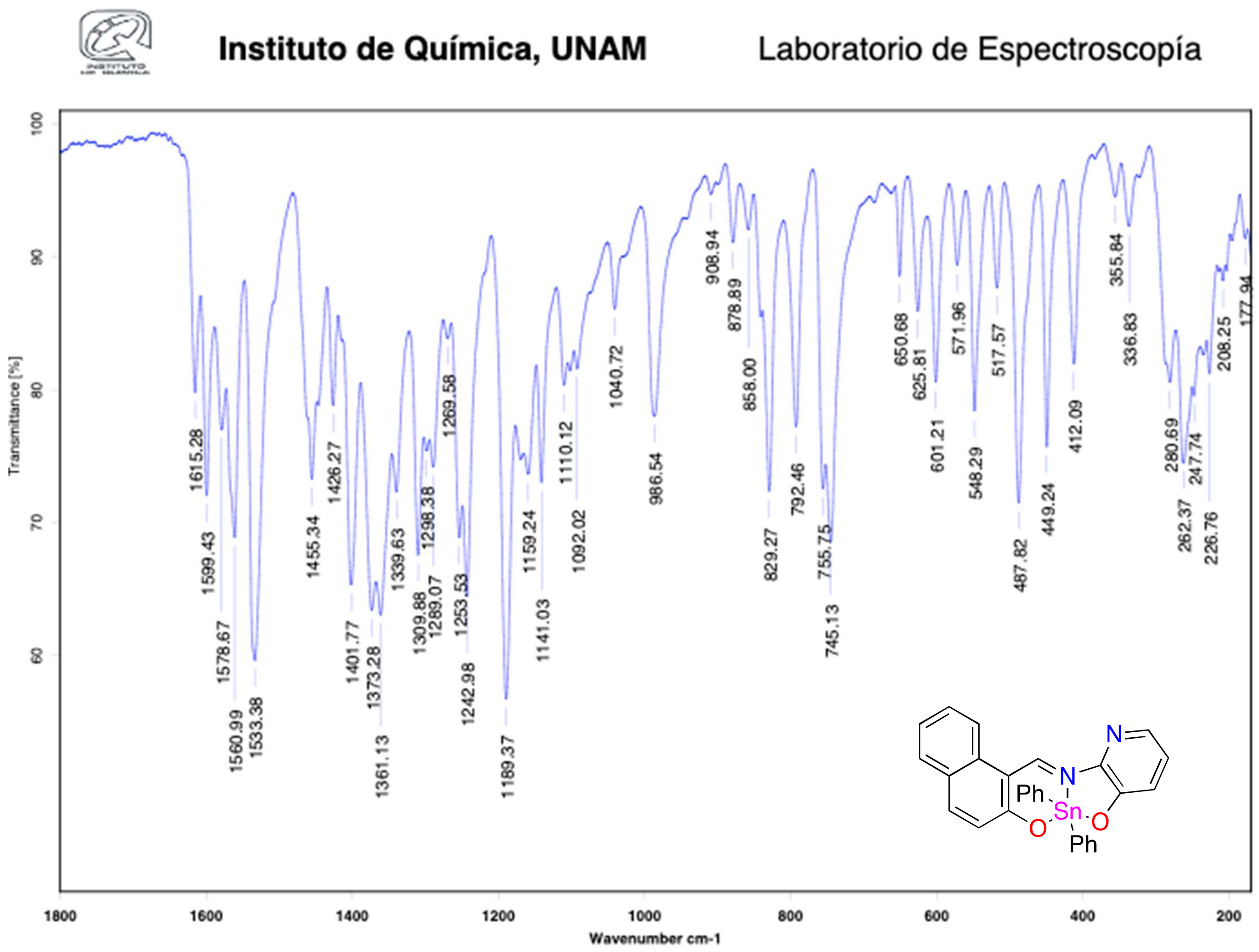

Figure S6.Far-IR of complex **1c**

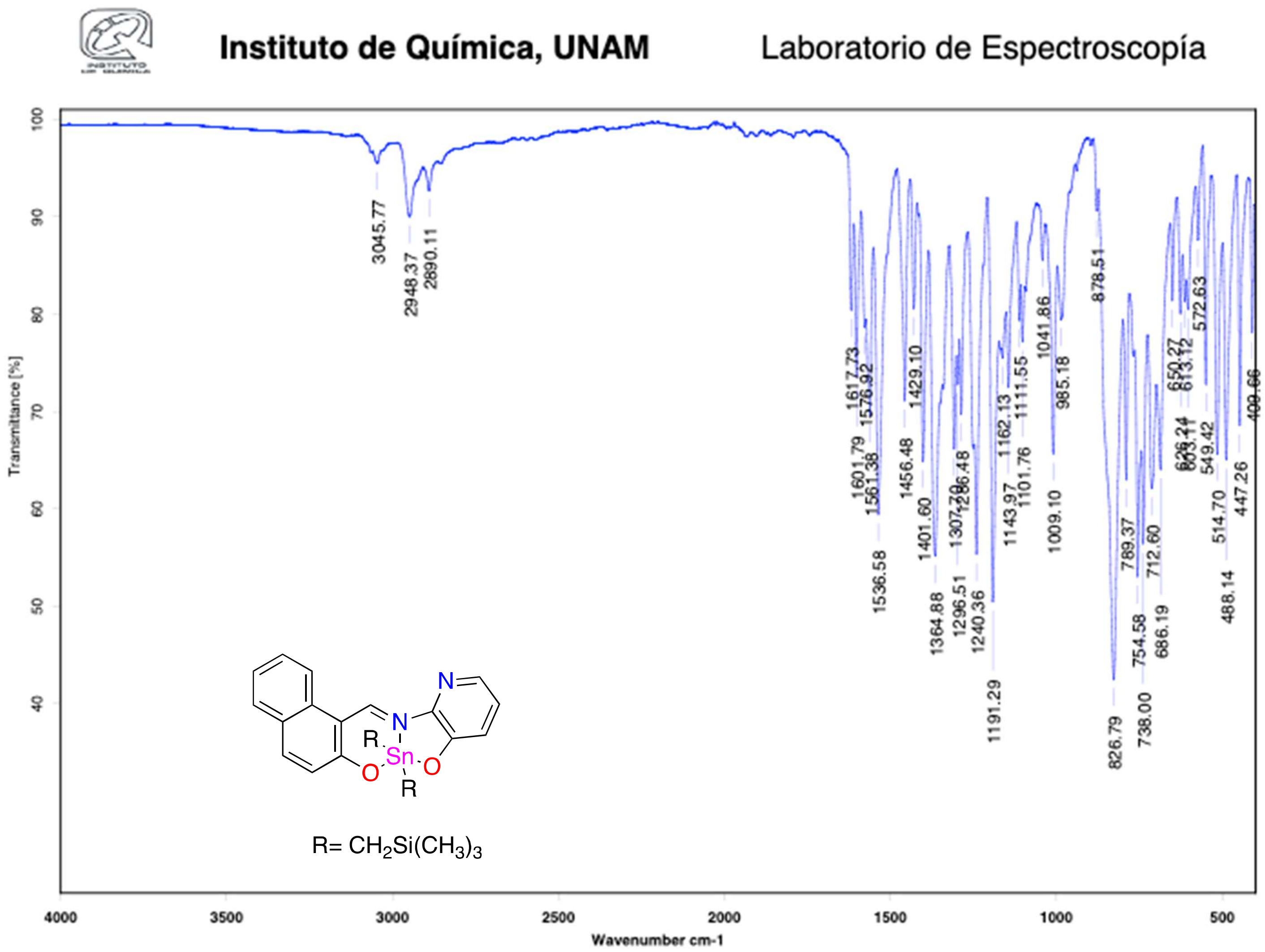

Figure S7. ATR-IR of complex **1d**

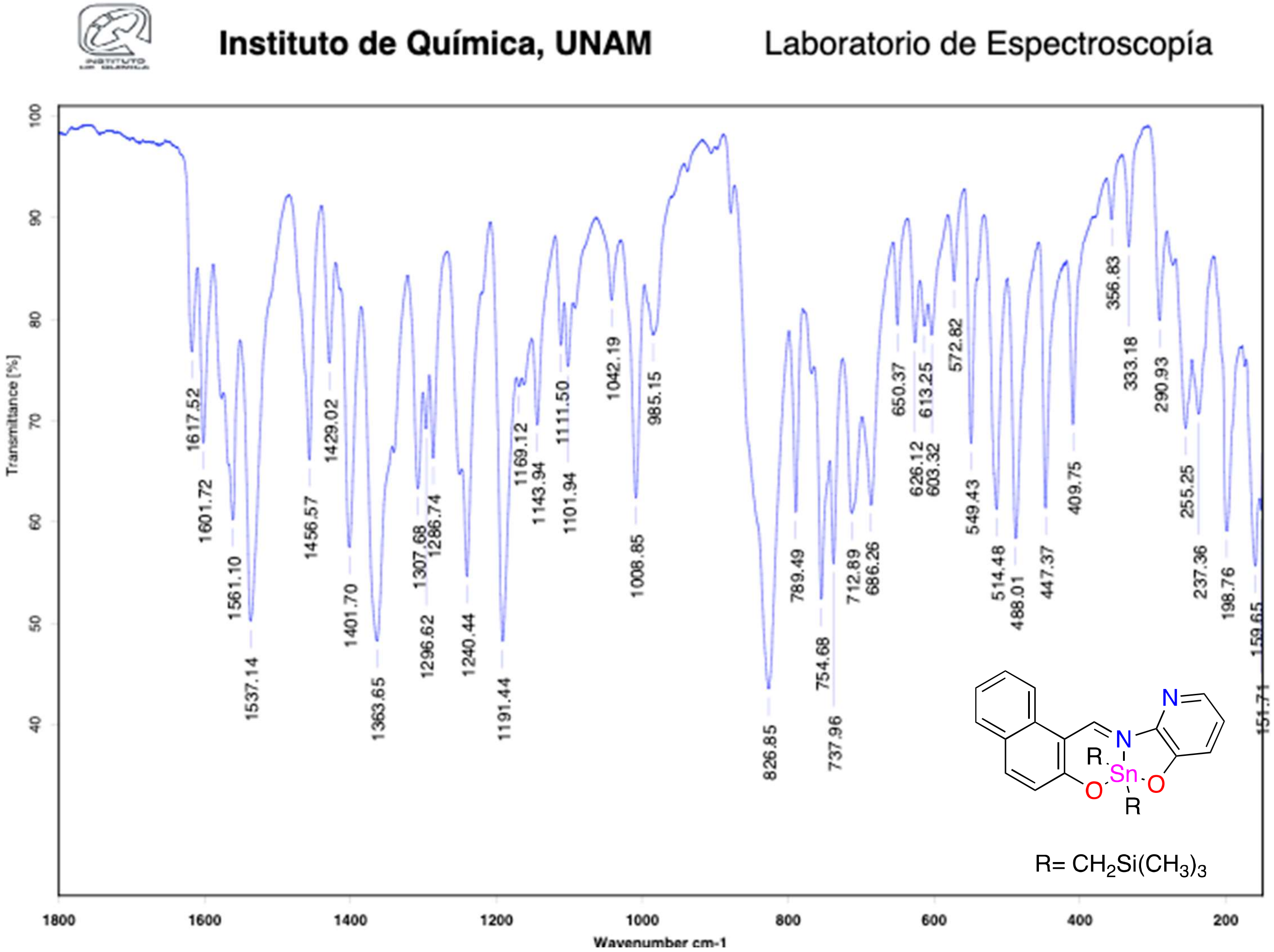

Figure S8. ATR-IR of complex **1d**

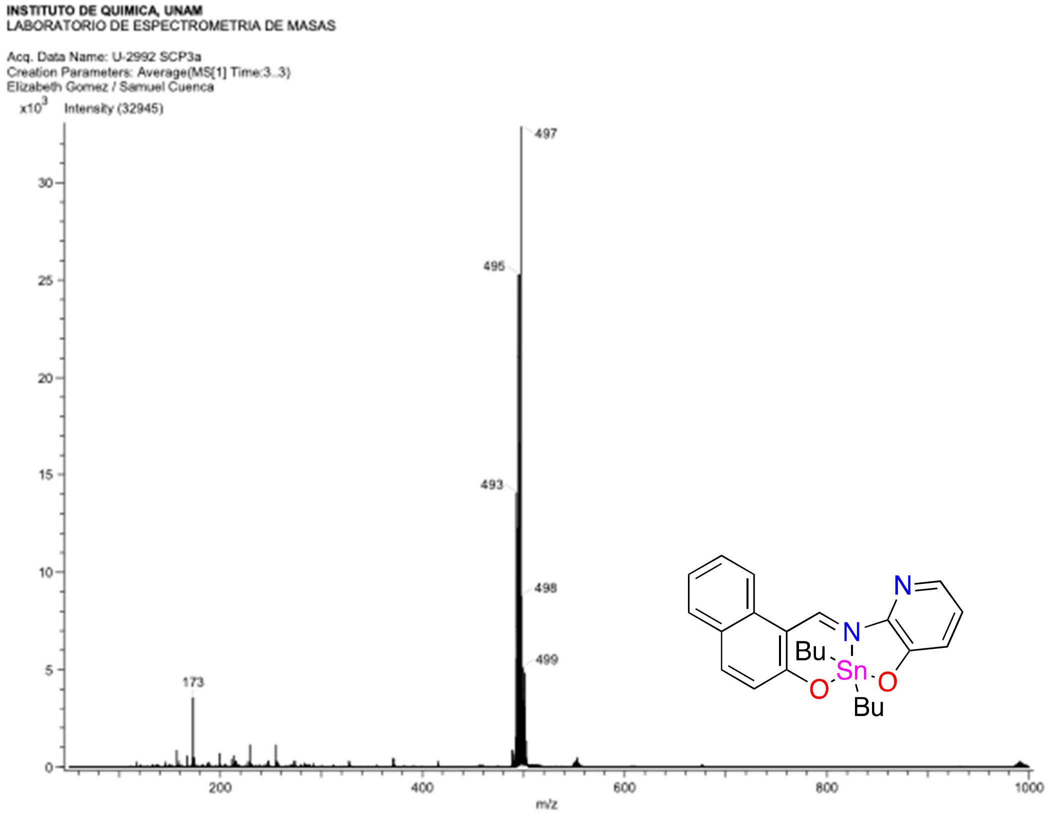

Figure S9. Mass Spectrometry (DART⁺) of complex **1a**

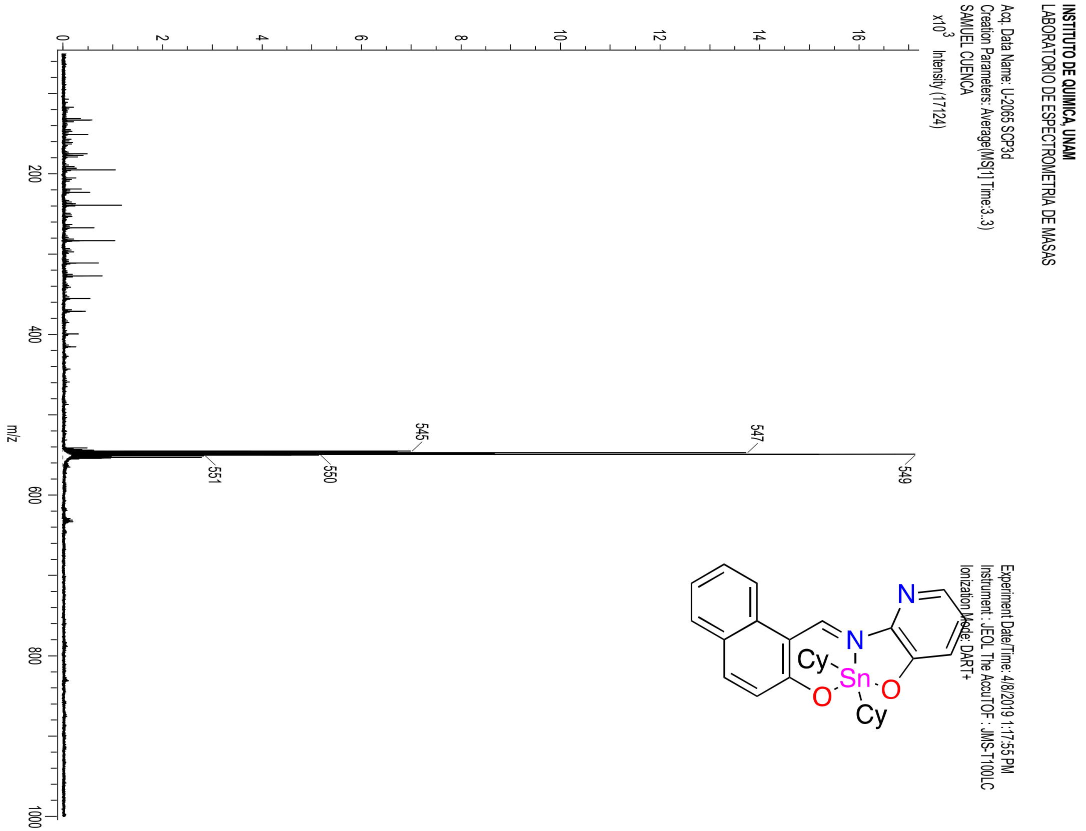

Figure S10. Mass Spectrometry (DART^{+}) of complex **1b**

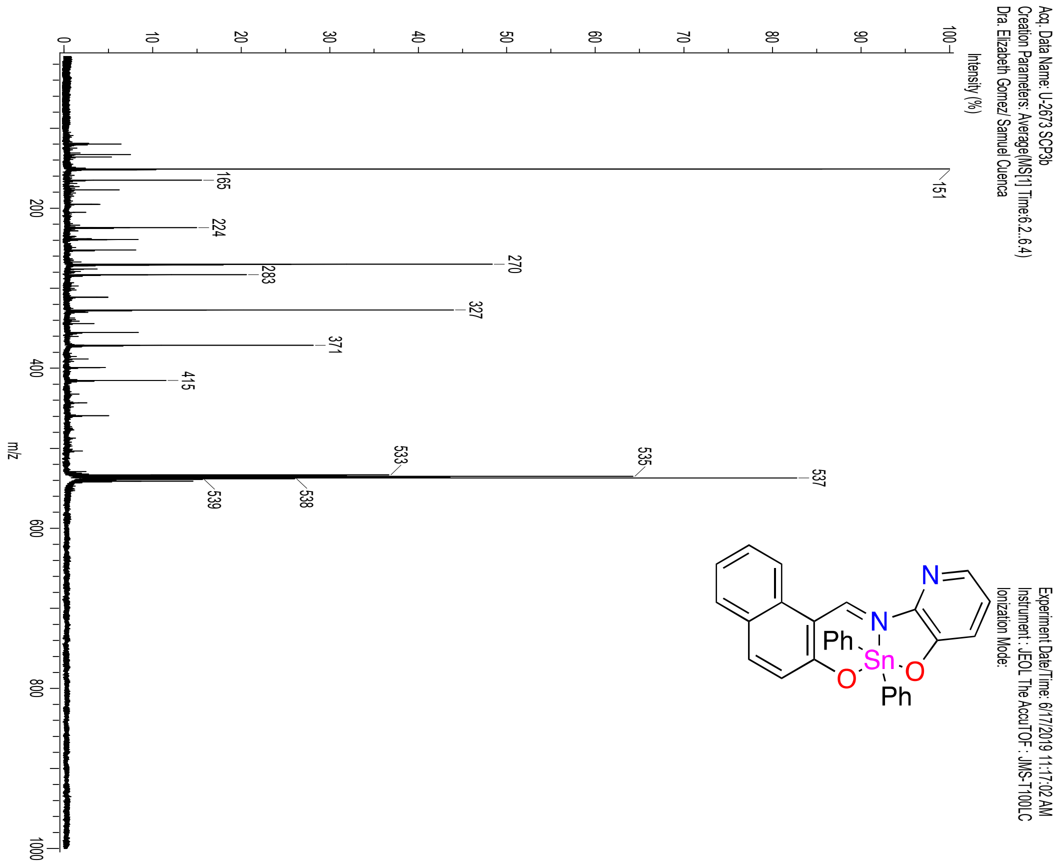

Figure S11. Mass Spectrometry (DART^{+}) of complex **1c**

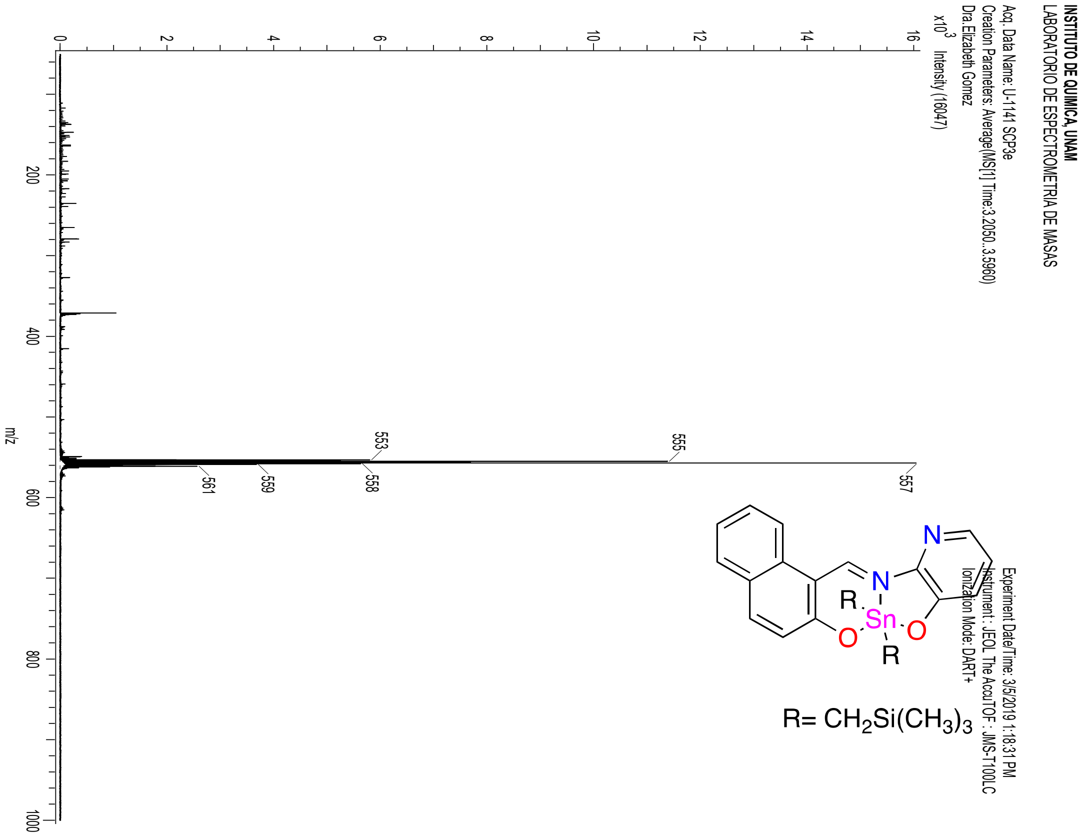

Figure S12. Mass Spectrometry (DART$^+$) of complex **1d**

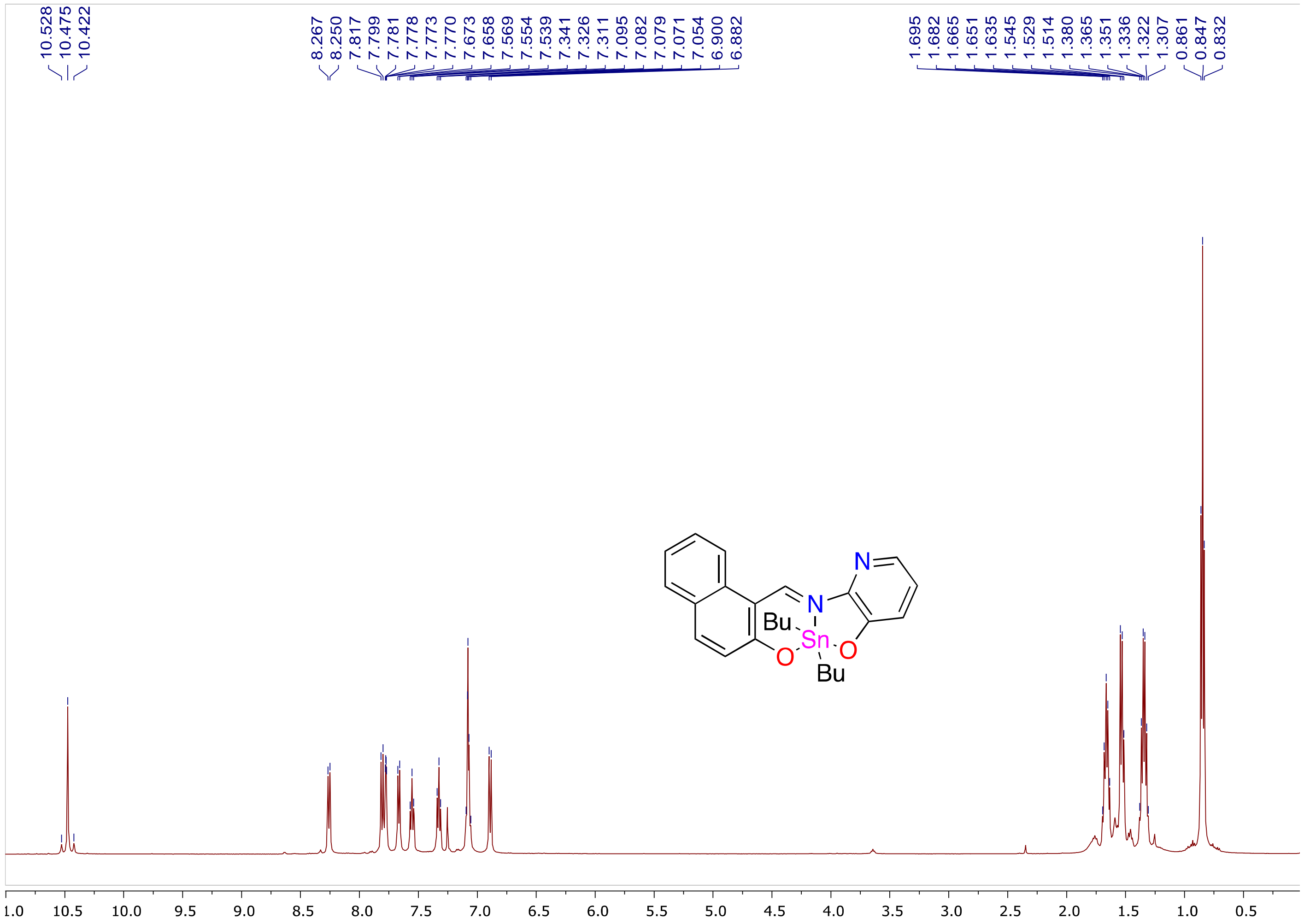

Figure S13. ^{1}H NMR ($CDCl_3$, 500 MHz) of complex **1a**

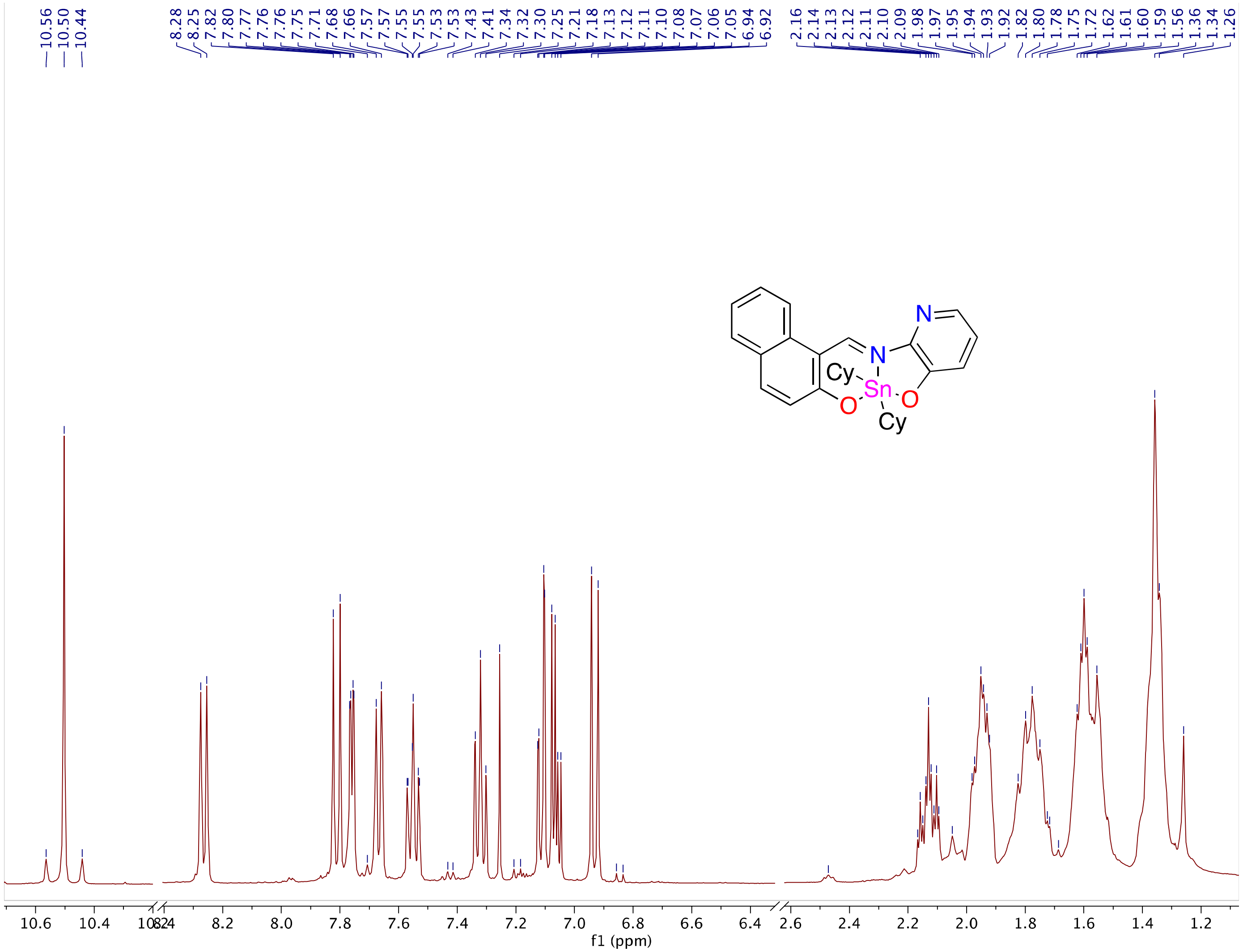

Figure S14. ^{1}H NMR ($CDCl_3$, 400 MHz) of complex **1b**

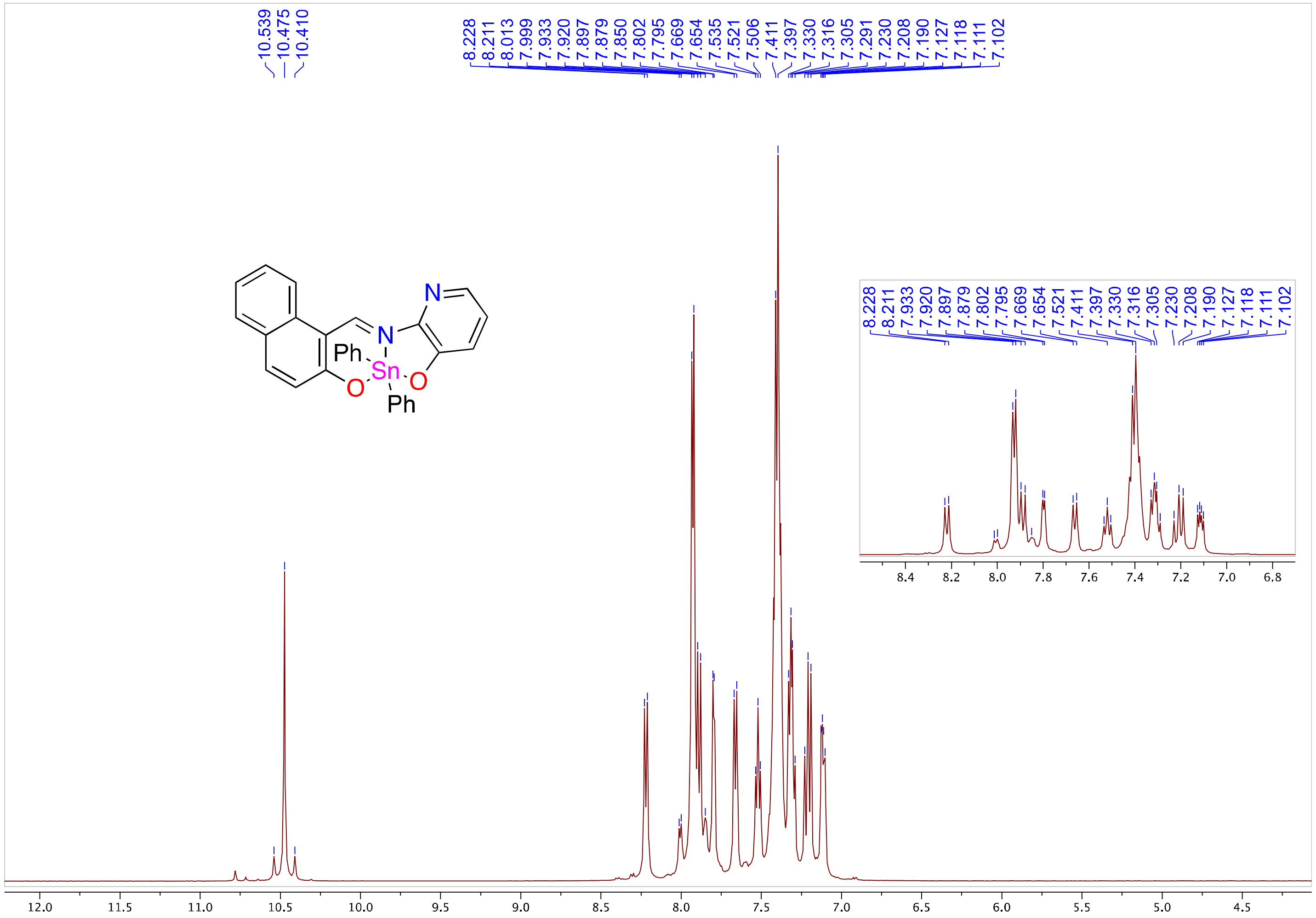

Fig. S15. ^{1}H NMR ($CDCl_3$, 500 MHz) of complex **1c**

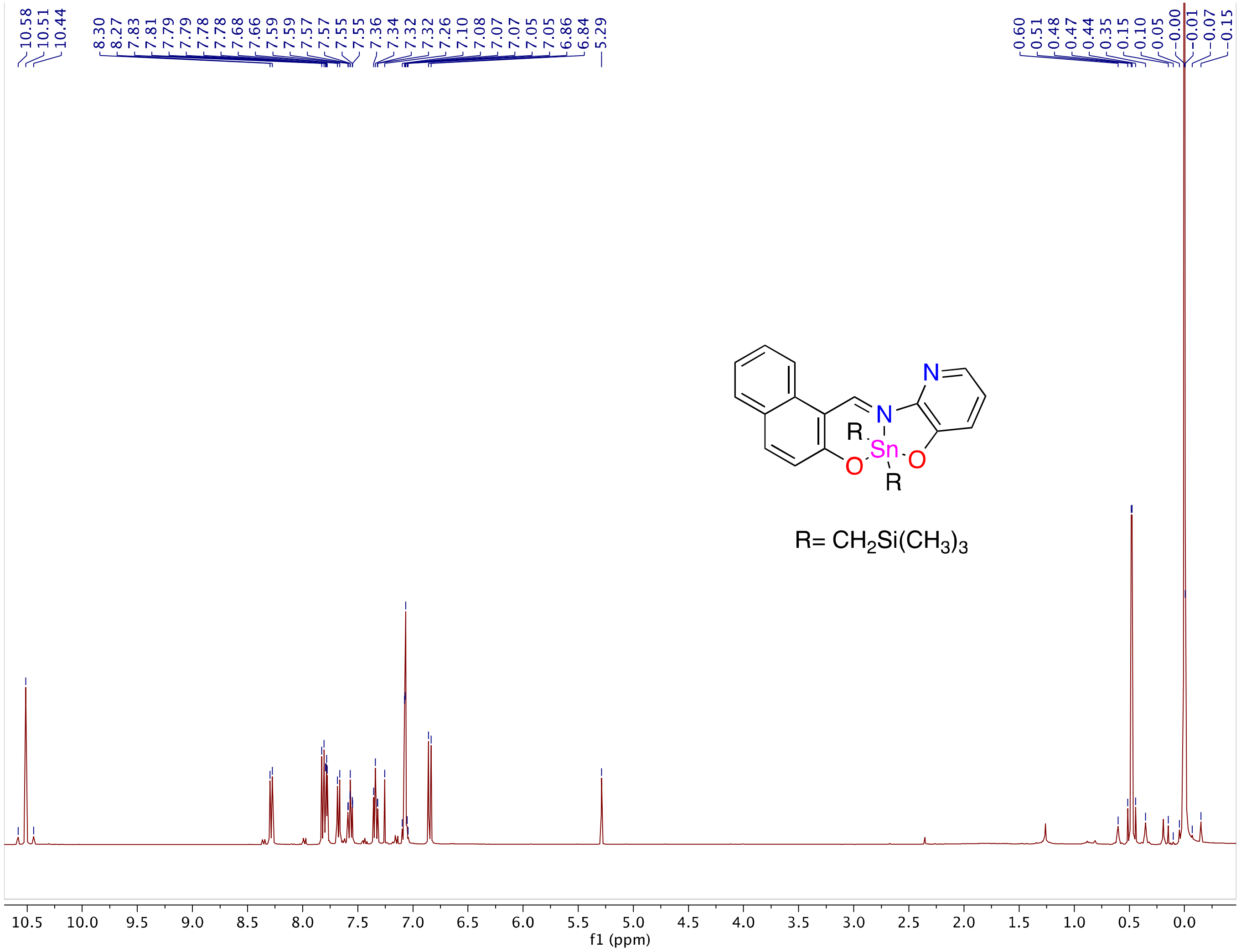

Figure S16. ^{1}H NMR ($CDCl_3$, 400 MHz) of complex **1d**

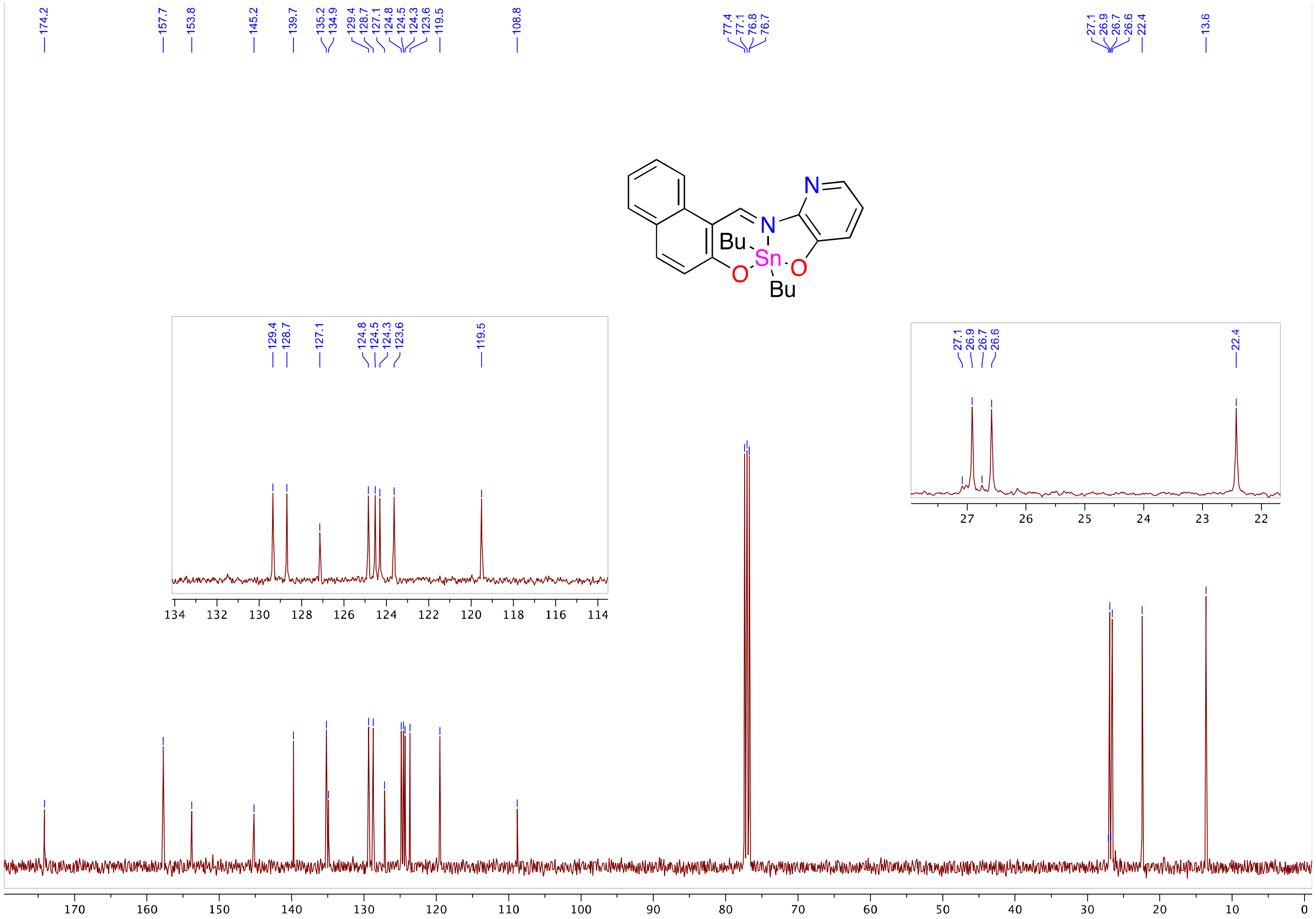

Figure S17. ^{13}CNMR ($CDCl_3$, 100 MHz) of complex **1a**

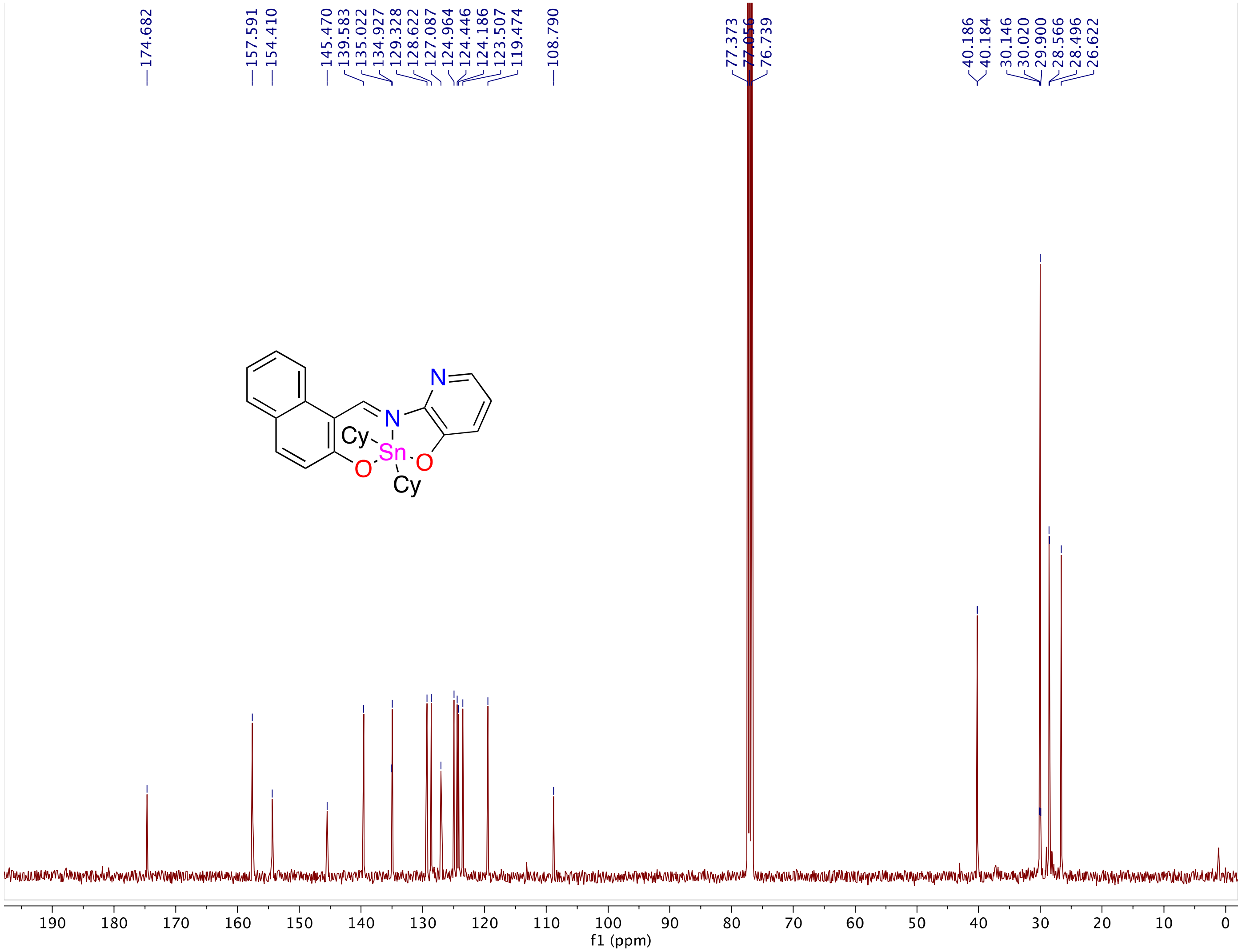

Figure S18. ^{13}CNMR ($CDCl_3$, 100 MHz) of complex **1b**

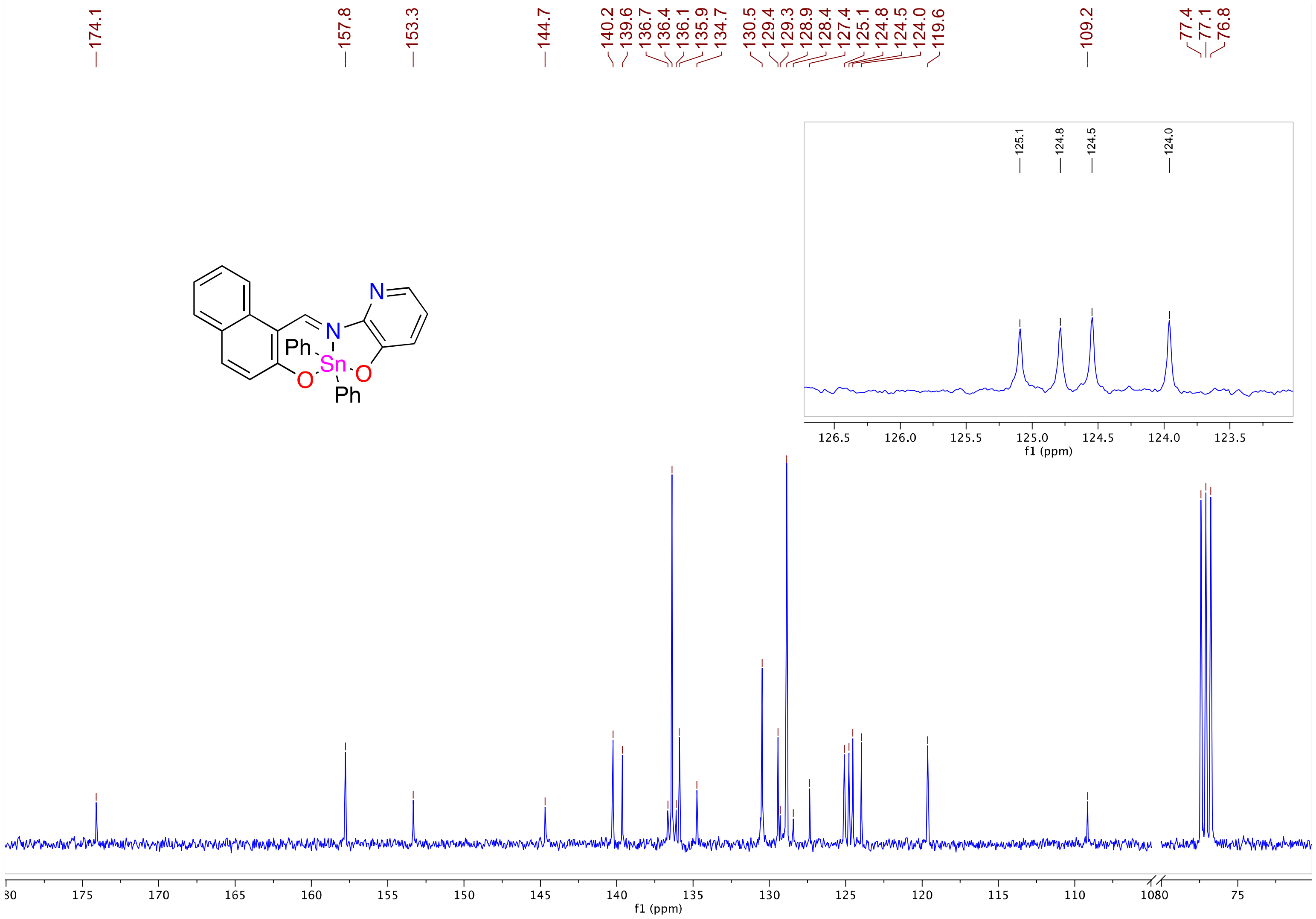

Figure S19. ^{13}C NMR ($CDCl_3$, 100 MHz) complex **1c**

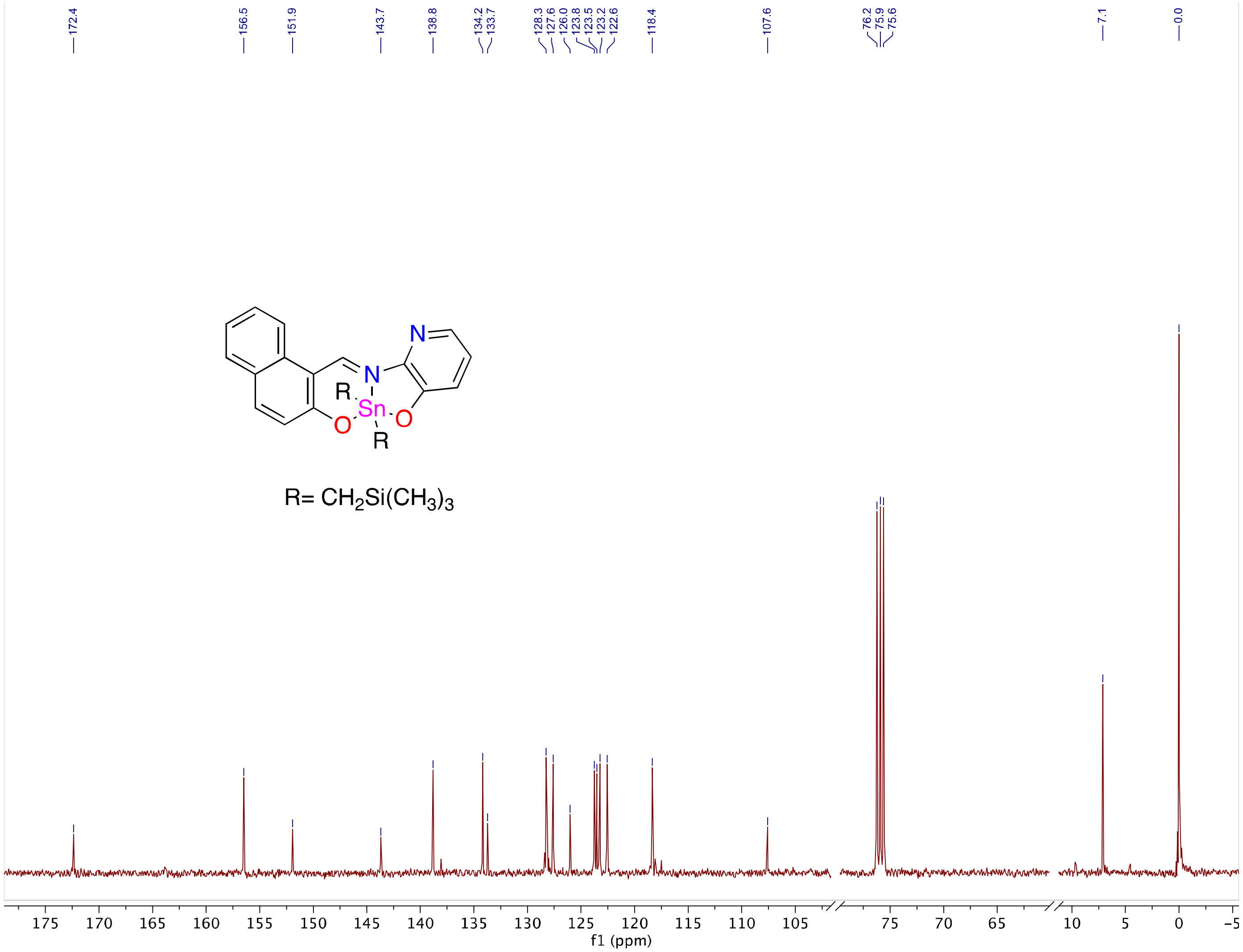

Figure S20. ^{13}C NMR ($CDCl_3$, 100 MHz) of complex **1d**

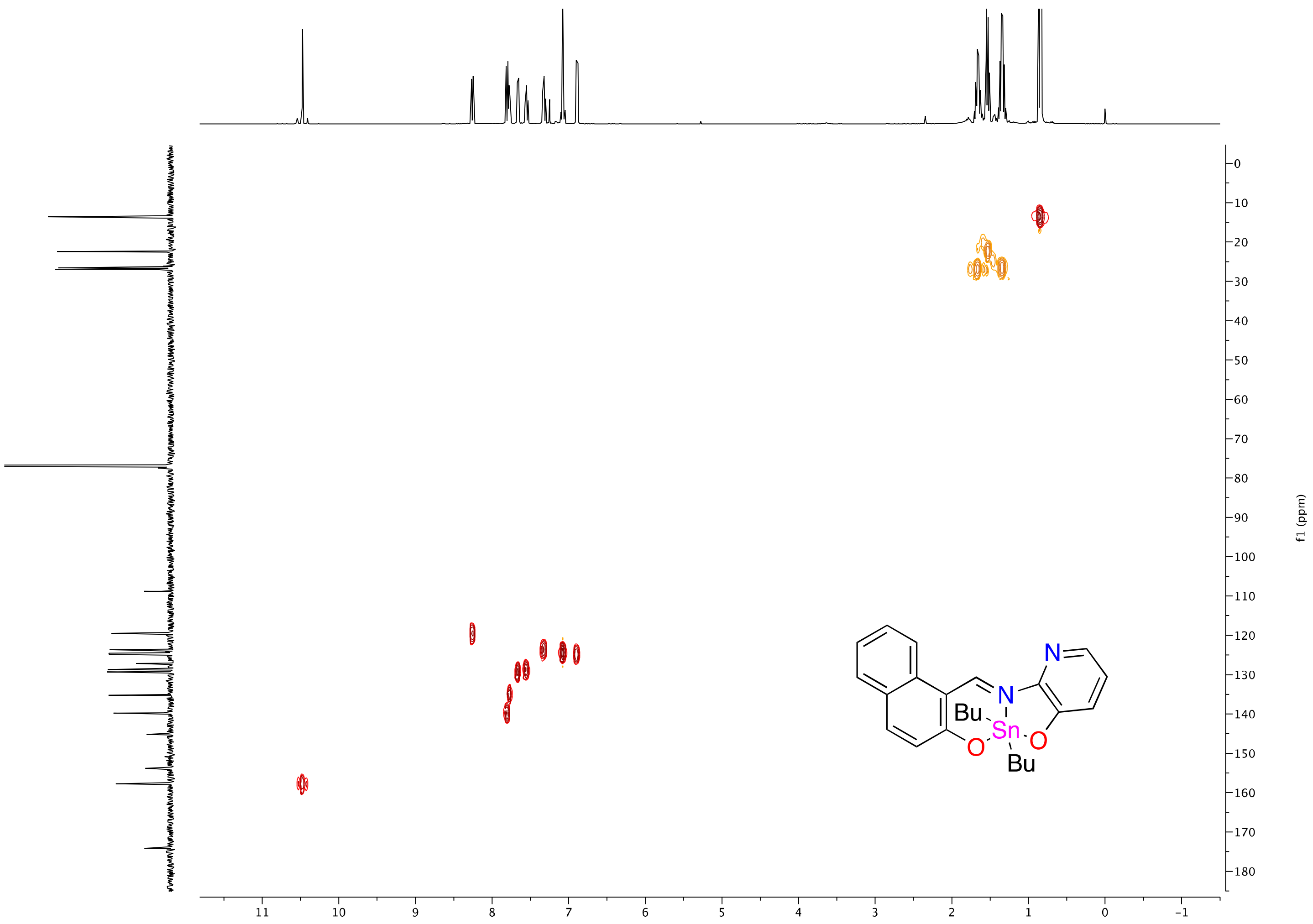

Figure S21. HSQC ($CDCl_3$) of complex **1a**

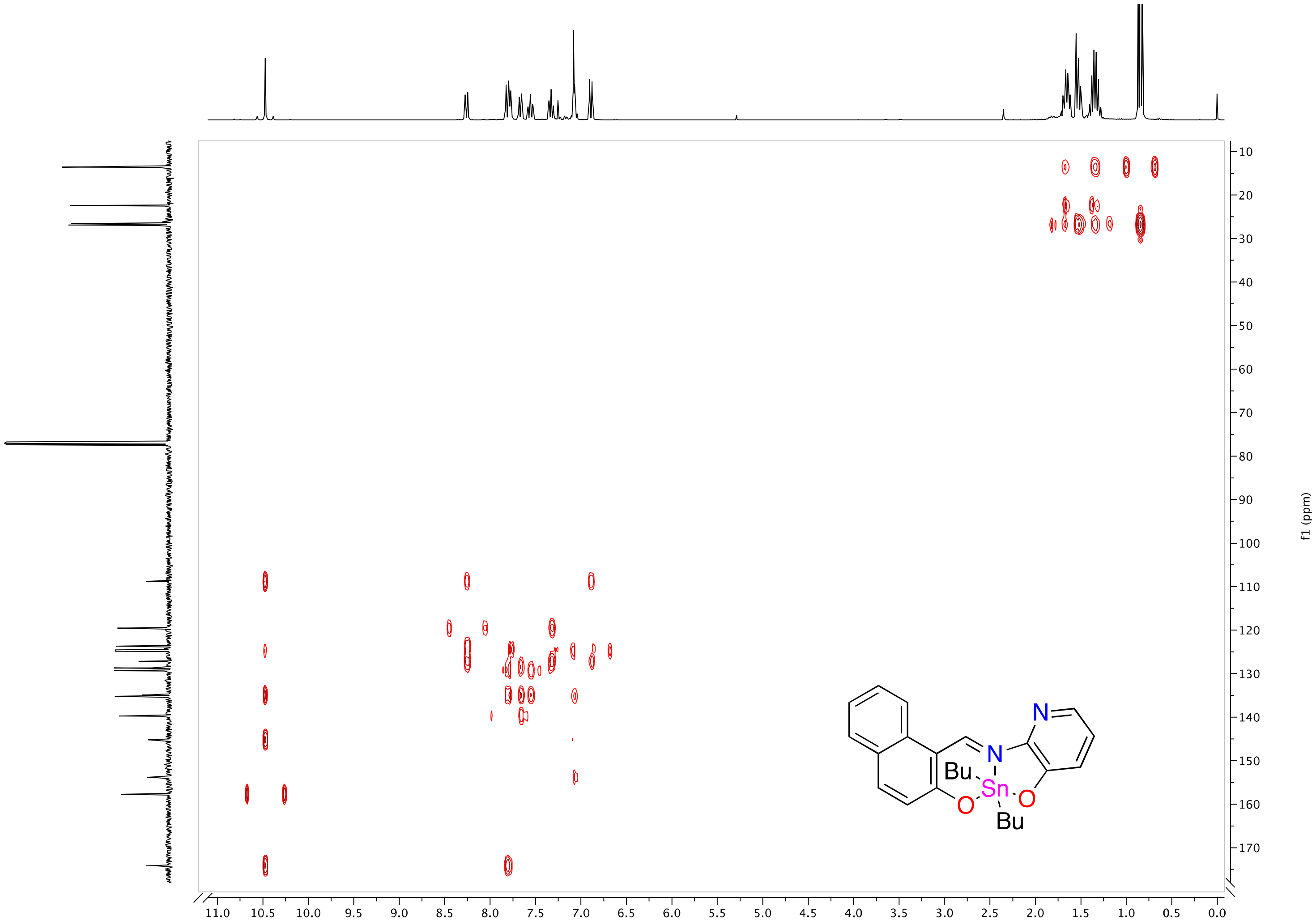

Figure S22. HMBC ($CDCl_3$) of complex **1a**

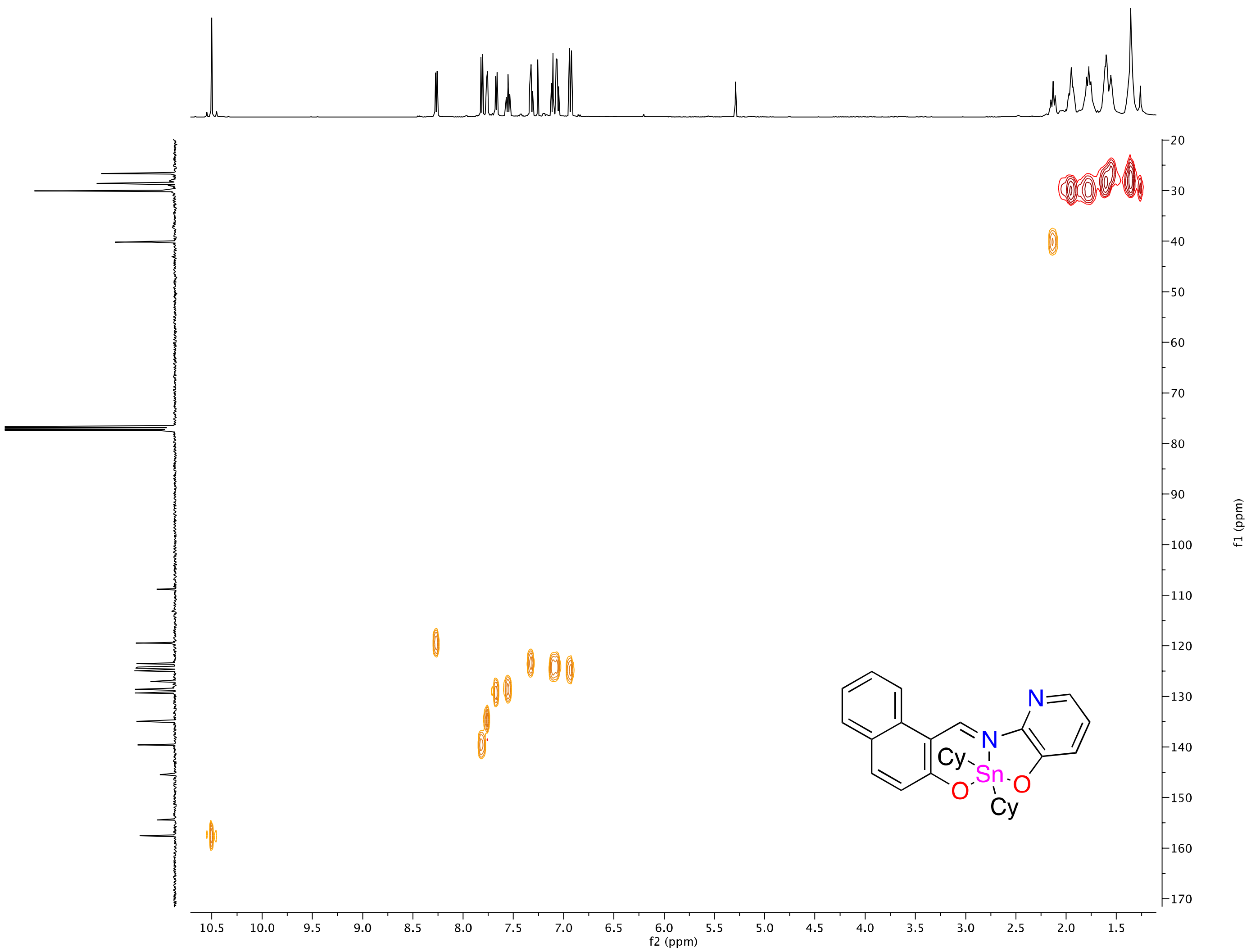

Figure S23. HSQC ($CDCl_3$) of complex **1b**

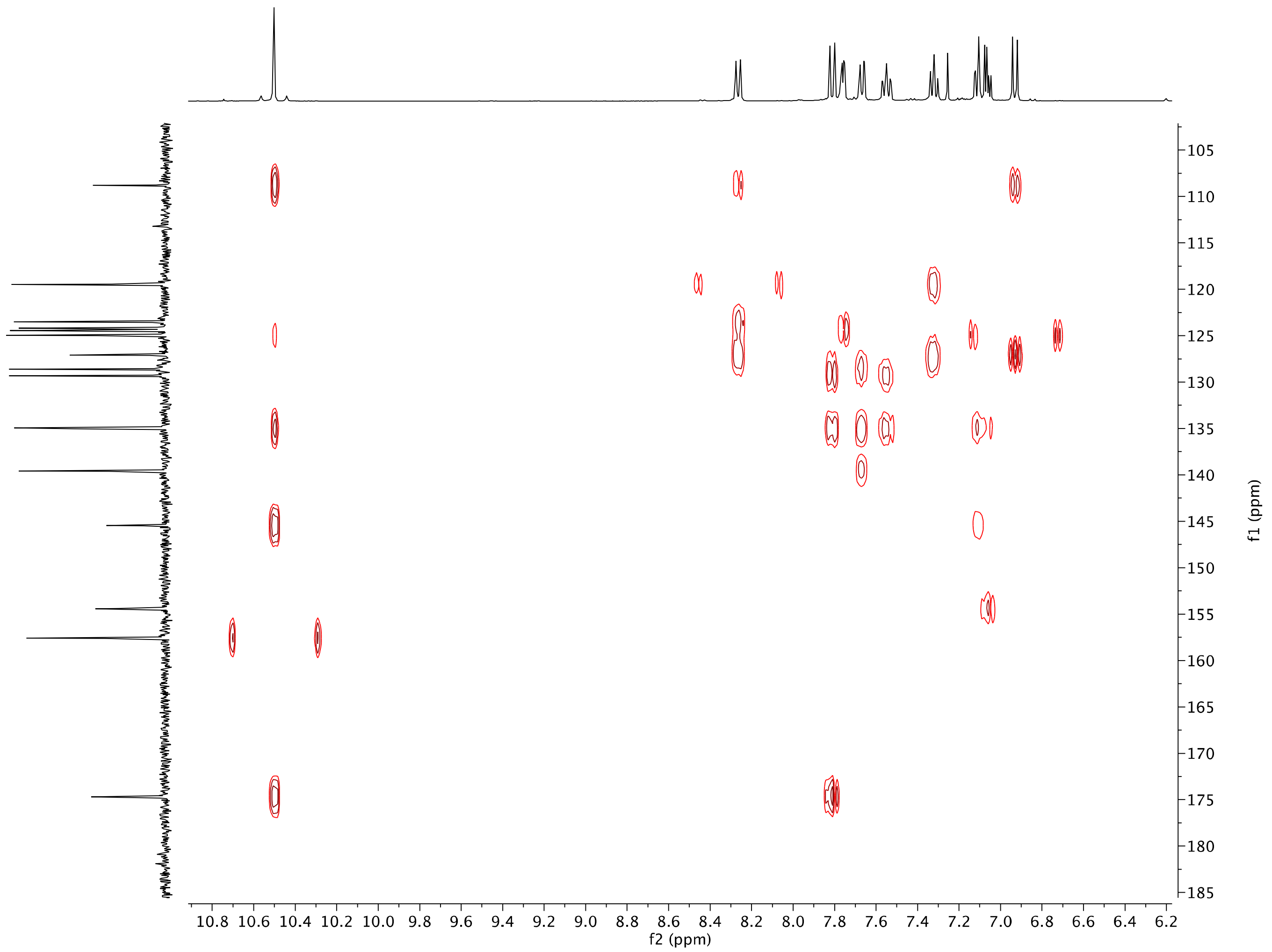

Figure S24. HMBC ($CDCl_3$) of complex **1b**

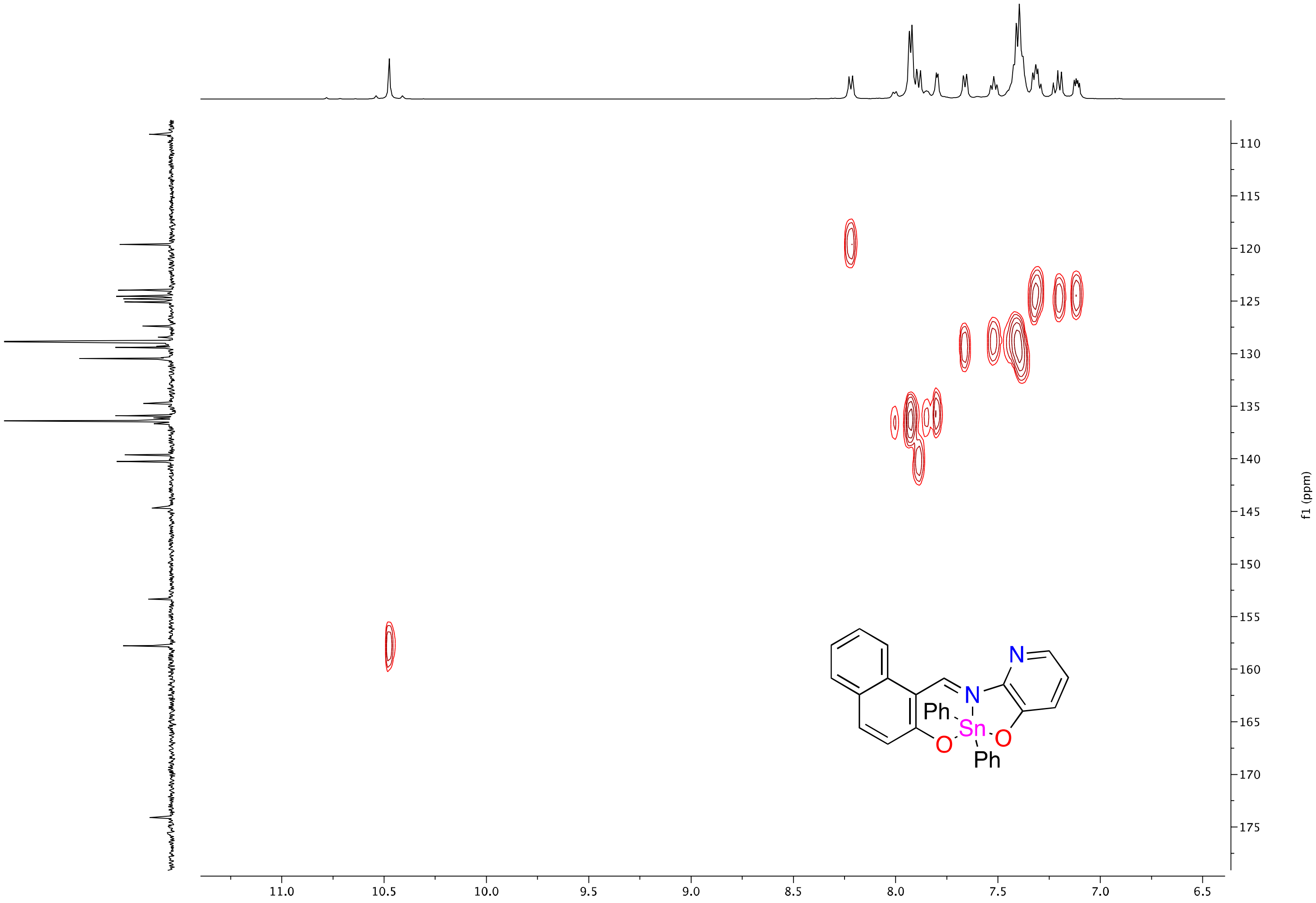

Figure S25. HSQC ($CDCl_3$) of compound **1c**

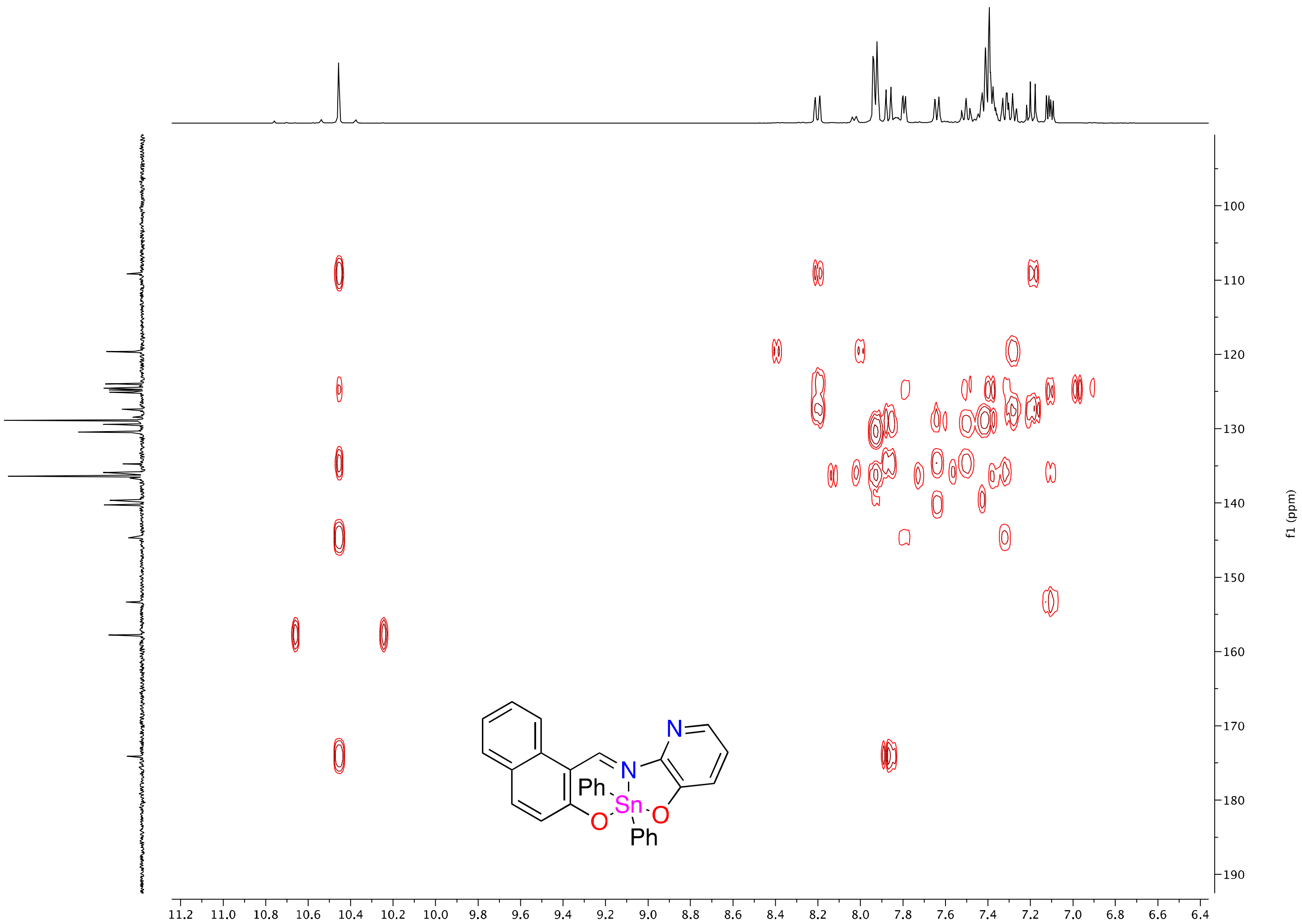

Figure S26. HMBC ($CDCl_3$) of compound **1c**

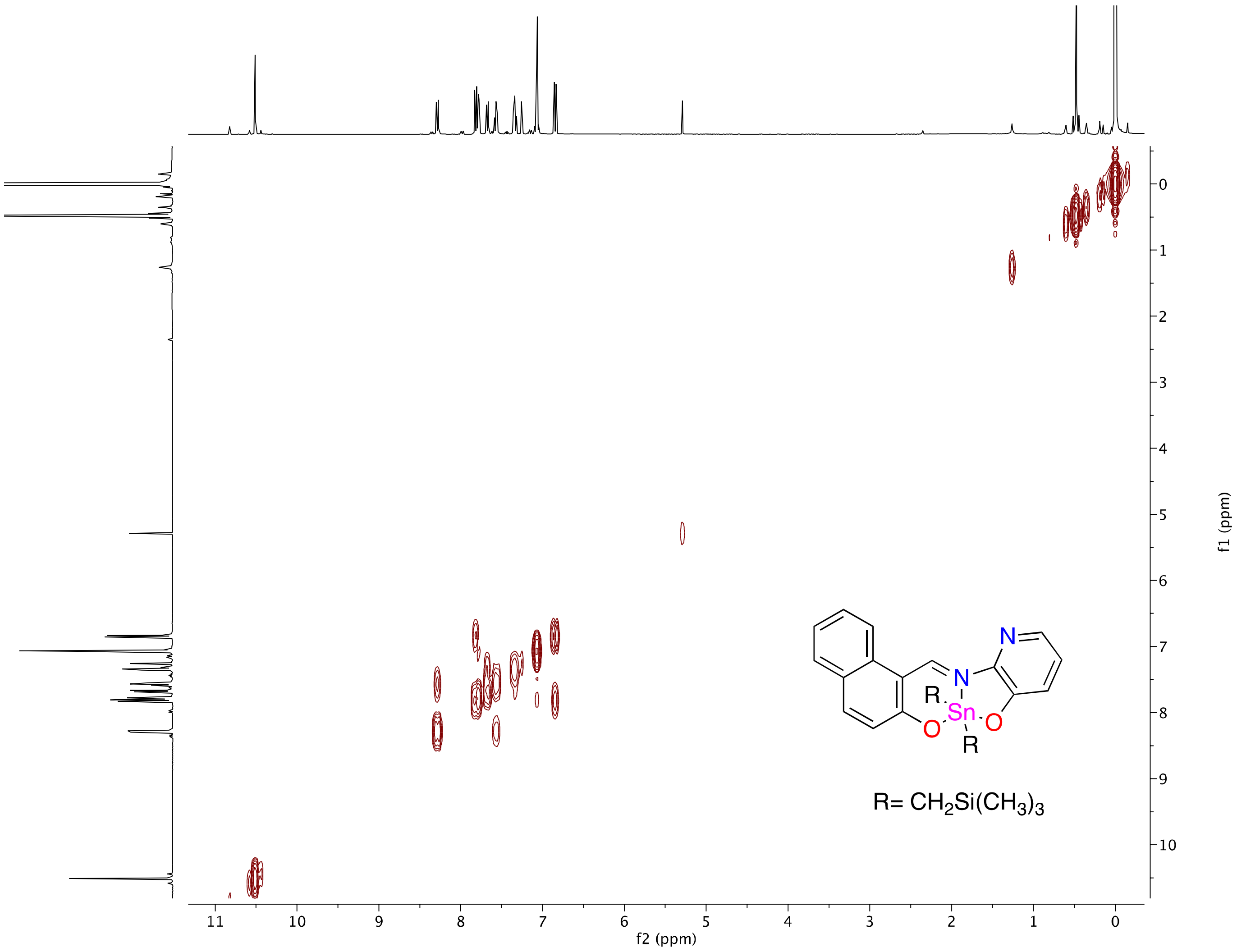

Figure S27. HSQC ($CDCl_3$) of complex **1d**

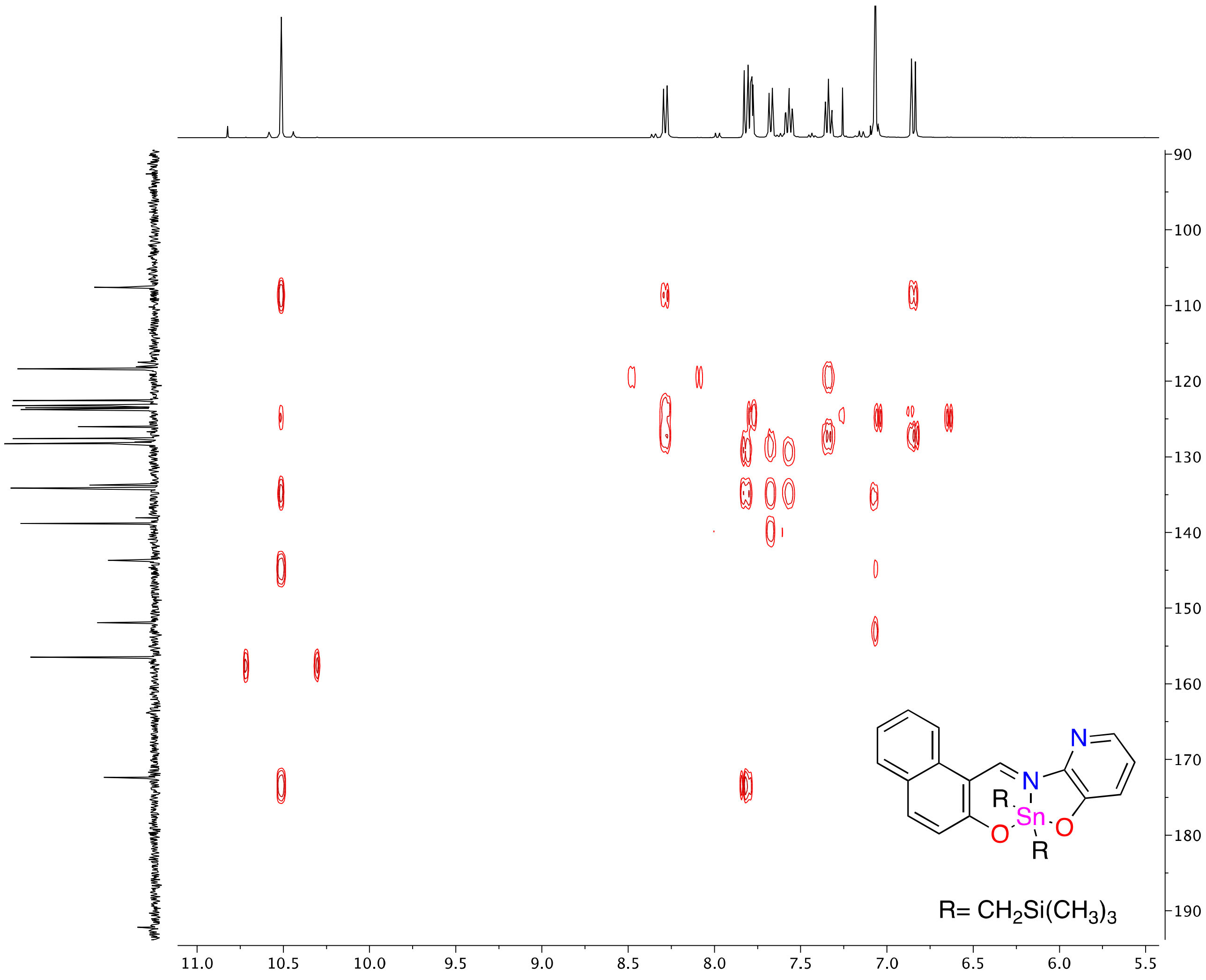

Figure S28. HMBC (CDCl$_3$) of complex **1d**

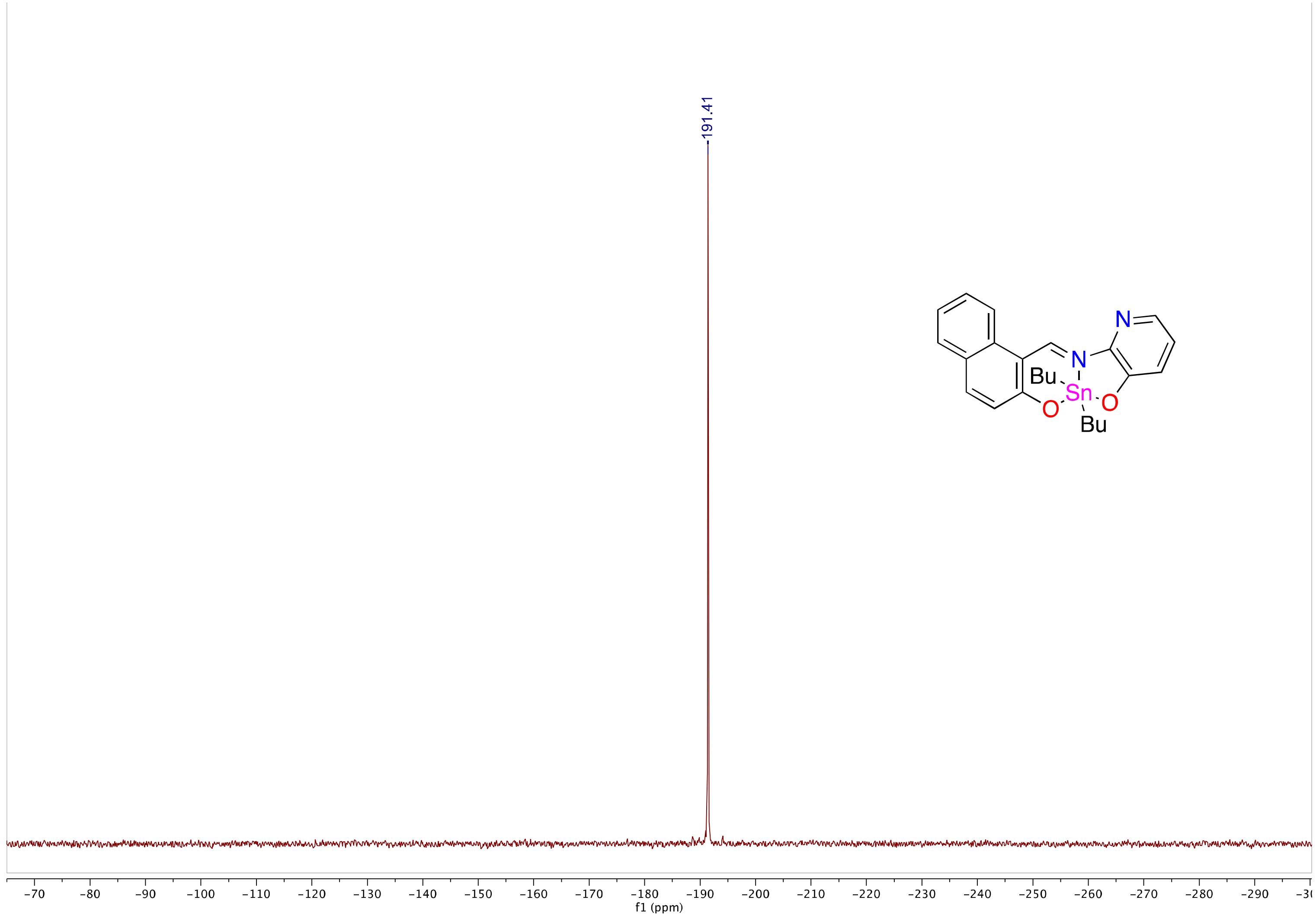

Figure S29. ^{119}Sn NMR ($CDCl_3$, 112 MHz) of complex **1a**

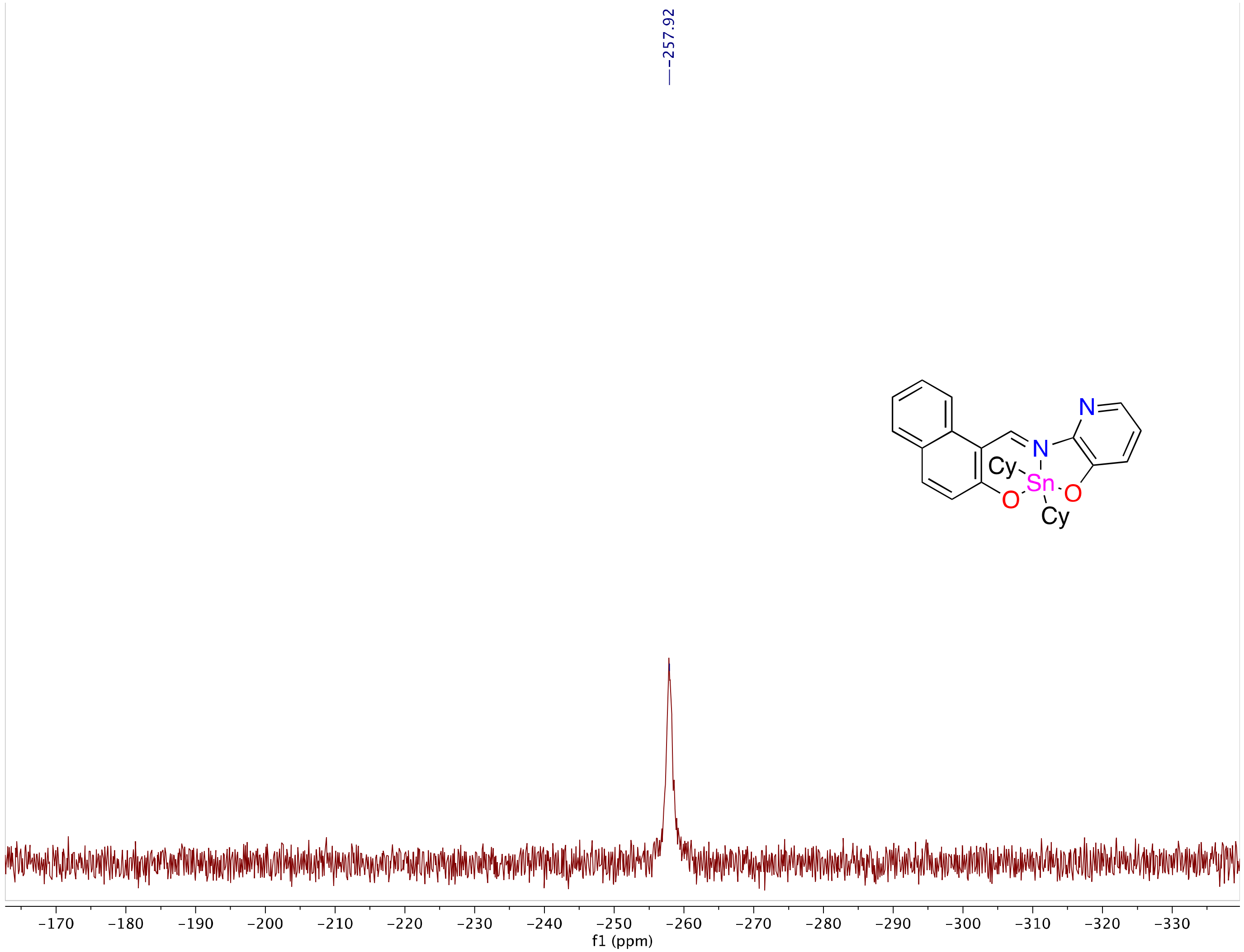

Figure S30. ^{119}Sn NMR ($CDCl_3$, 112 MHz) of complex **1b**

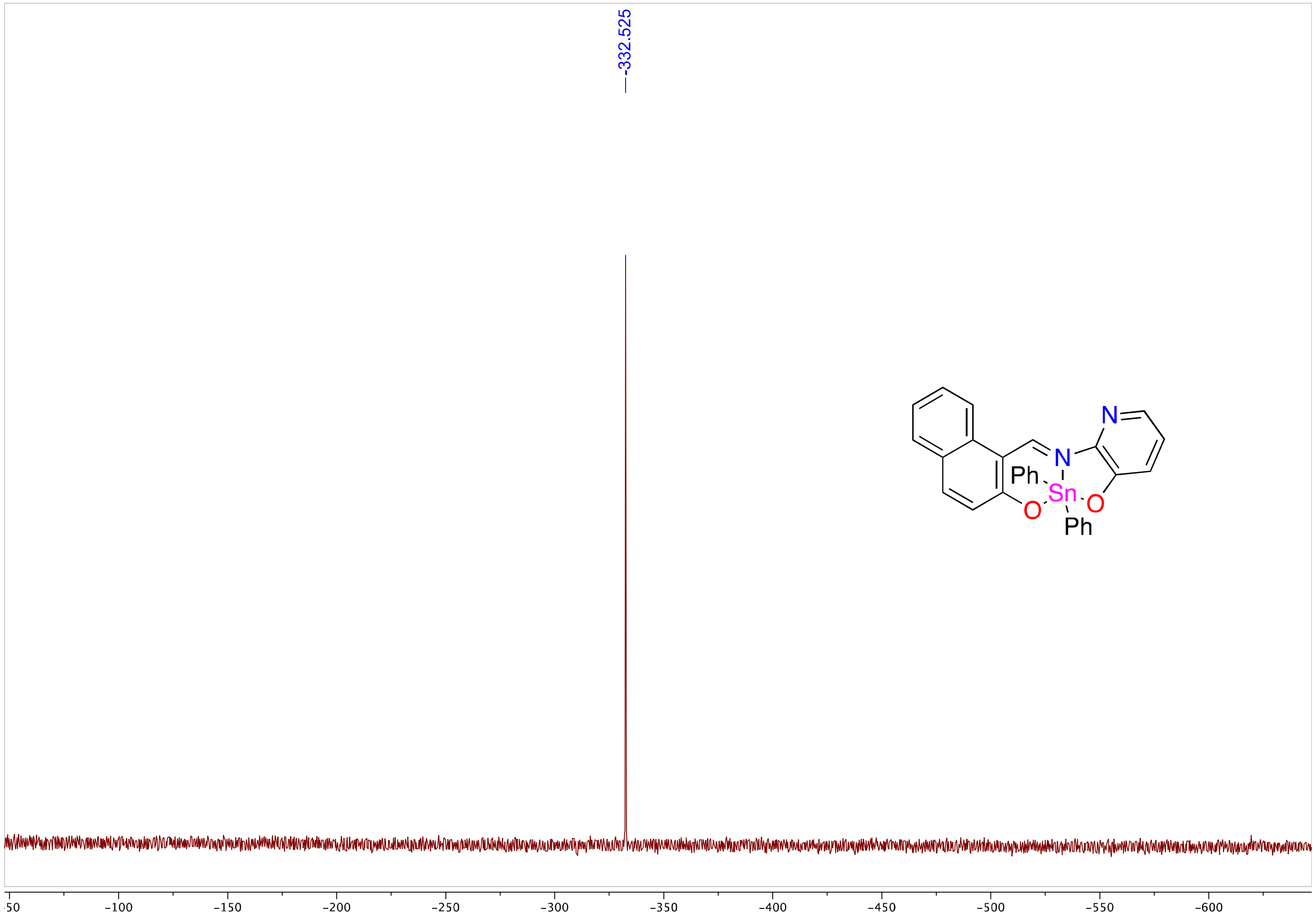

Figure S31. ^{119}Sn NMR ($CDCl_3$, 112 MHz) of complex **1c**

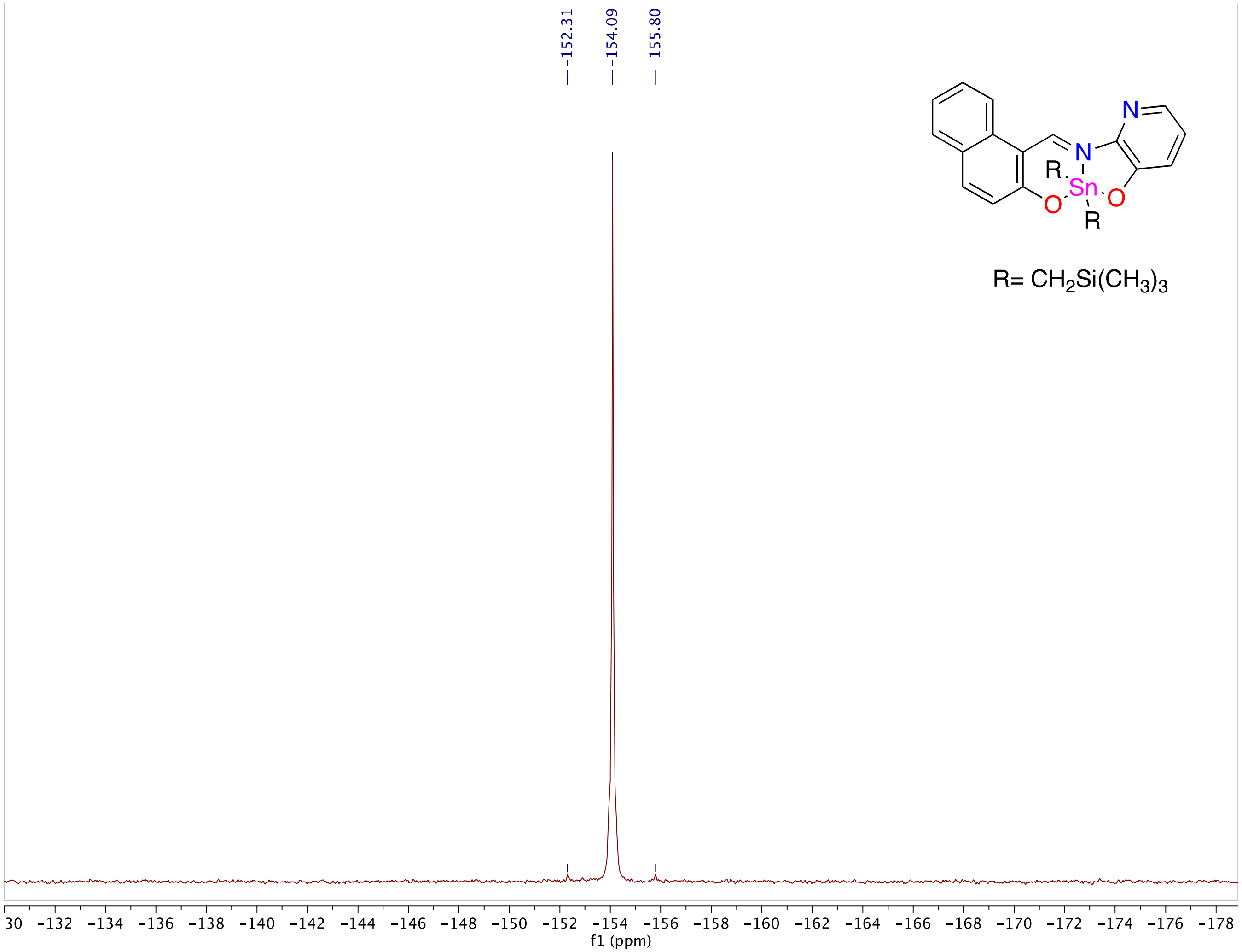

Figure S32. ^{119}Sn NMR ($CDCl_3$, 112 MHz) of complex **1d**
